# The effectiveness of non-pharmacological sleep interventions for people with chronic pain: a systematic review and meta-analysis

**DOI:** 10.1186/s12891-022-05318-5

**Published:** 2022-05-11

**Authors:** Katie Whale, Jane Dennis, Vikki Wylde, Andrew Beswick, Rachael Gooberman-Hill

**Affiliations:** 1 Musculoskeletal Research Unit, Translational Health Sciences, Bristol Medical School, University of Bristol, Learning and Research Building, Level 1, Southmead Hospital, Bristol, BS10 5NB UK; 2 National Institute for Health Research Bristol Biomedical Research Centre, University Hospitals Bristol and Weston NHS Foundation Trust and the University of Bristol, Bristol, UK

**Keywords:** Chronic pain, Sleep, Sleep interventions, psychological, Systematic review, Meta-analysis

## Abstract

**Objective:**

About two thirds of people with chronic pain report problems sleeping. We aimed to evaluate the effectiveness of non-pharmacological sleep interventions for improving sleep in people with chronic pain.

**Design:**

We conducted a systematic review of non-pharmacological and non-invasive interventions to improve sleep quality or duration for adults with chronic non-cancer pain evaluated in a randomised controlled trial. Our primary outcome of interest was sleep; secondary outcomes included pain, health-related quality of life, and psychological wellbeing. We searched the Cochrane Library, MEDLINE, Embase, PsycINFO and CINAHL from inception to April 2020. After screening, two reviewers evaluated articles and extracted data. Meta-analysis was conducted using a random effects model. Risk of bias was assessed with the Cochrane tool.

**Results:**

We included 42 trials involving 3346 people randomised to 94 groups, of which 56 received an intervention targeting sleep. 10 studies were of fair and 32 of good methodological quality. Overall risk of bias was judged to be low in 11, high in 10 and unclear in 21 studies. In 9 studies with 385 people randomised, cognitive behavioural therapy for insomnia showed benefit post-treatment compared with controls for improved sleep quality, standardised mean difference − 1.23 (95%CI -1.76, − 0.70; *p* < 0.00001). The effect size was only slightly reduced in meta-analysis of 3 studies at low risk of bias. The difference between groups was lower at 3 and 6 months after treatment but still favoured cognitive behavioural therapy for insomnia. Pain, anxiety and depression were reduced post-treatment, but evidence of longer term benefit was lacking. There was no evidence that sleep hygiene interventions were effective in improving sleep and there was some evidence in comparative studies to suggest that cognitive behavioural therapy for insomnia was more effective than sleep hygiene.

Numerous other interventions were evaluated in small numbers of studies, but evidence was insufficient to draw conclusions about effectiveness.

**Conclusions:**

Cognitive behavioural therapy for insomnia is an effective treatment to improve sleep for people with chronic pain, but further high-quality primary research is required to explore combined CBT content that will ensure additional improvements to pain, quality of life and psychological health and longer-term maintenance of benefits. Primary research is also needed to evaluate the effectiveness of interventions for which insufficient evidence exists.

**Trial registration:**

PROSPERO registration number: CRD42019093799.

**Supplementary Information:**

The online version contains supplementary material available at 10.1186/s12891-022-05318-5.

## Background

While chronic pain may be a primary complaint or secondary to an underlying disease, it is now recognised as a health condition in its own right [1] requiring specific therapy and rehabilitation [2]. In the UK, chronic pain affects between a third and a half of the population and about 10–14% of people report moderate to severely disabling chronic pain [3]. For people affected and their families, chronic pain is associated with a reduced quality of life and impacts on work and social life [4–6]. About two thirds of people with chronic pain report problems sleeping [7], including difficulties falling asleep, staying asleep, or waking early [6, 8–10], and this is evident across a range of conditions associated with chronic pain. In a large US population, 89% of people with chronic pain caused by fibromyalgia reported one, and 63% reported two or more symptoms of sleep disturbance [11]. Sleep disorders are common in people with multiple sclerosis [12, 13], rheumatoid arthritis [14], and osteoarthritis [15–17], with about 60–75% of people affected. Sleep disturbance is greater in people with more severe osteoarthritis symptoms [15, 18]. Other pain conditions with associated sleep disturbance include migraine and frequent headache [19–21], and low back [8, 22] and neck pain [23].

The relationship between sleep and pain is bidirectional [24–27]. Reduced sleep leads to greater pain, and greater pain has a negative impact on sleep. Poor sleep is also associated with the development of chronic pain [24]. In a large Norwegian cohort, women with three symptoms of insomnia (problems falling asleep, waking early and work disruption) were nearly three times more likely to develop fibromyalgia compared with those with no symptoms [28]. In addition, chronic sleeping difficulties are a predictor of acute post-operative pain in patients undergoing total knee replacement [29]. From the other direction, studies have demonstrated that reduced sleep is causally linked to greater pain [26, 30, 31], increasing both the neurotransmitters related to pain sensitivity and the inflammatory markers associated with pain [32, 33]. Restricted total sleep time and frequent waking, similar to the sleep patterns experienced by those with chronic pain, results in high spontaneous pain reports and reduced pain modulation. Improving sleep for people with chronic pain therefore has the potential to reduce pain levels and improve quality of life. The aim of this study was to use systematic review methods and meta-analysis to evaluate the effectiveness of non-pharmacological sleep interventions in improving sleep in people with chronic pain.

## Methods

The protocol was registered prospectively with PROSPERO (CRD 42019093799) [34], and the research question formulated according to the PICO principle [35]. Methods were based on those described in the Cochrane Handbook [36], and reporting was in accordance with Preferred Reporting Items for Systematic Reviews and Meta-Analyses (PRISMA) guidelines [37]. (Supplement Table 1).

### Patient and public involvement

All of our studies of sleep problems in people with chronic pain are fully supported by patient involvement. This includes regular discussions during development, conduct and reporting of research.

### Eligibility criteria

Eligible studies reflected PICOS criteria:

- Population: People aged ≥18 years with chronic non-cancer pain
- Intervention: Non-pharmacological and non-invasive intervention to improve sleep quality or duration
- Comparison: Comparator of standard care, no treatment, attentional, or wait list control
- Outcomes: Primary outcomes of sleep quality and duration, secondary outcomes of other sleep outcomes, pain, health-related quality of life, and psychological wellbeing, and a primary harm outcome of adverse events. Follow up post-treatment and at 3 and 6 months after end of treatment if reported
- Study: Evaluation in a randomised controlled trial

### Information sources and searches

We searched MEDLINE, EMBASE, PsycINFO, Cochrane Library, and CINAHL from inception up to 8th April 2020. The search strategy as applied in MEDLINE is included in Supplement Table 2. Citations of key reviews and studies were tracked in Web of Science, reference lists checked and clinical trial records reported in the Cochrane Library followed up. No language restrictions were applied, and relevant non-English articles were translated. Studies reported only as abstracts or that we are unable to acquire using inter-library loans or email contact with authors were excluded.

### Study screening and data extraction

Results of searches were imported into Endnote and duplicates removed. After an initial screen by one reviewer to remove clearly off-topic studies, all titles and abstracts were screened independently by two reviewers. Potentially relevant articles were acquired and independently assessed by two reviewers for eligibility with disagreements resolved in discussion with a third reviewer.

One reviewer extracted data from eligible studies into Excel and a second reviewer checked this. Extracted data comprised: country; dates of recruitment; setting; inclusion and exclusion criteria; participant characteristics (chronic pain condition, age, sex); intervention and comparator content, timing, duration and intensity; assessment times; outcome measures; and information on intervention fidelity. We contacted study authors for clarification relating to review eligibility and for missing data.

### Risk of bias assessment

Risk of bias was assessed independently by two reviewers using the Cochrane tool [36], specifically relating to: randomisation process; deviations from intended interventions; missing outcome data (> 20% considered high risk), measurement of the outcome; and selection of the reported result. Studies with serious concerns relating to risk of bias were considered high risk and those with limited reporting unclear risk. Studies with wait list controls were considered to be at unclear risk of bias due to inherent lack of blinding. Studies with high or unclear risk of bias were excluded from meta-analysis in sensitivity analysis.

### Data synthesis

We conducted meta-analyses with Review Manager 5.4 software to compare outcomes across studies with similar interventions and outcome measures. For continuous data, if outcomes were measured identically across studies, an overall mean difference (MD) with 95% confidence intervals (CIs) was calculated. If continuous outcomes were measured differently across studies, overall standardised mean differences (SMDs) and 95% CIs were calculated and presented alongside measures of heterogeneity (I^2^). Forest plots were generated. Risk of bias as a potential source of heterogeneity was considered in sensitivity analyses. If pooling of outcome data was not appropriate, a narrative synthesis was reported. In interpreting the outcomes from this review we consider effect sizes as described by Cohen: small, SMD = 0.2; medium, SMD = 0.5; large, SMD = 0.8 [38].

## Results

**Fig. 1:**
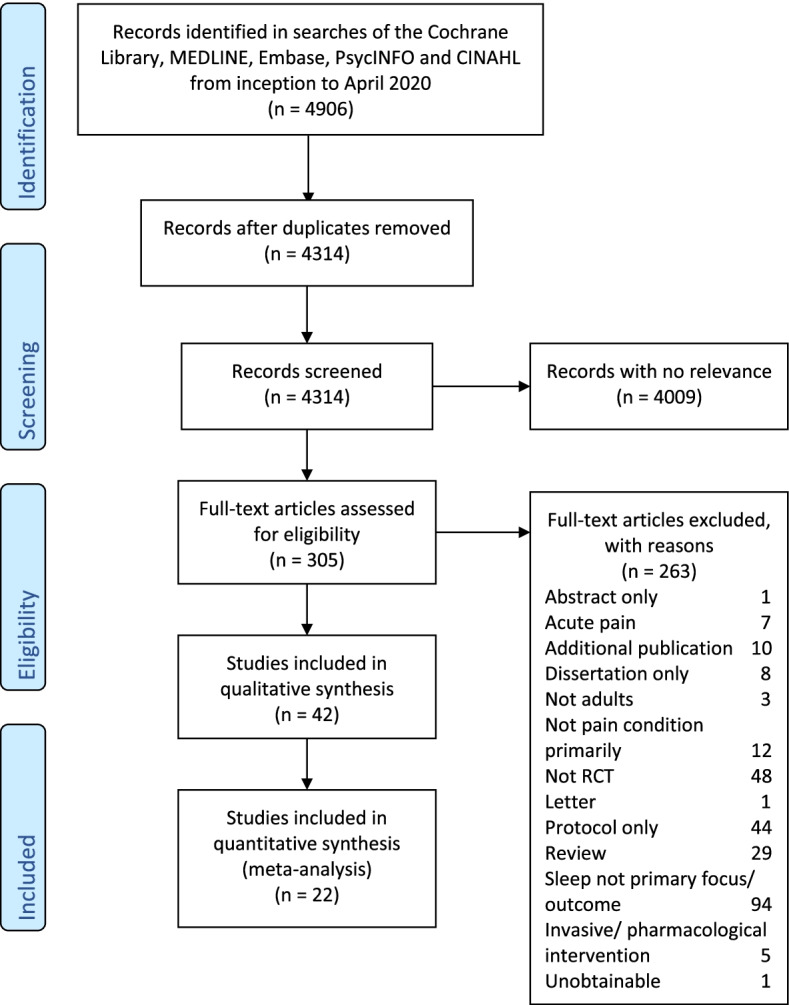
Study flow diagram

Review progress is summarised in Fig. 1. Searches identified 4314 articles of which 305 were considered potentially eligible. After detailed screening we included 42 randomised trials which included 3346 participants. In 33 trials, 2 randomised groups were compared, in 8 there were 3 groups and in 1 there were 4. Overall, 94 groups were compared.

Details of studies and methodological quality scores are summarised in Supplement Table 3 and risk of bias assessments in Supplement Table 4. Methodological quality was assessed as good in 32 studies and fair in ten. Overall risk of bias was judged to be low in 11, high in ten and unclear in 21 studies. The mean ages of participants in studies ranged from 31 to 73 years, with an average age across studies of 51 years. Overall, 56 groups received an intervention targeting improvements to sleep. Country of study, chronic pain condition, intervention characteristics, and outcome measures are summarised in Table 1.

**Table 1:** Study and intervention characteristics

Author Country, Recruitment dates Setting Study design	Inclusion Number randomised (intervention; control) Age % female Common treatment	Intervention/ control Number of sessions/ duration Therapist Fidelity		Follow up Outcomes Losses to follow up Risk of bias issues Methodological quality score^a^ Key results
**1. Psychological interventions**				
**1.a. Cognitive behavioral therapy focusing on insomnia compared with control**				
Abbasi et al. 2016 [39] Iran, 2014 Setting not specified Parallel group RCT	Multiple sclerosis, at least 6 months from time of diagnosis, score of 5 or more for PSQI quality of sleep 72 (36; 36) Mean age 34 years 100% female	CBT targeting sleep quality 8 weekly 90 min sessions Psychiatric nurse Fidelity not reported Group sessions talking about feelings and experiences. Treatment with “common drugs” 3 sessions Therapist not described		Before, immediately and 1 month after treatment PSQI. No pain, HRQoL, psychological health measures. Adverse events not reported. Overall loss to follow up 8% Unclear risk of bias as selected outcomes reported and methodological detail of trial limited. 19/28 Mean score of sleep quality of patients in the intervention group had a significant difference at 3 stages of before, immediately and 1 month after the intervention.
Currie et al. 2000 [40] Canada, dates not stated Group Parallel group RCT	Chronic musculoskeletal pain (excluding fibromyalgia) and sleep difficulties, age < 60 years 60 (32; 28) Mean age 45 years 55% female	CBT. Sleep and insomnia education, behavioural therapy, relaxation training, cognitive restructuring, sleep hygiene education. Coping with chronic pain sleep problems manual. 7 × 2 h group sessions held once a week for 7 weeks Clinical psychology doctoral students or interns with training in CBT. Structured manual. Regular supervision Waiting list controls		Baseline, end of treatment (7 weeks), 3 months Sleep diaries. Actigraphy measured nocturnal activity levels. PSQI. Multidimensional Pain Inventory Pain Severity scale, no HRQoL measure, BDI. Adverse events not reported. Overall loss to follow up 15% at 3 months Unclear risk of bias due to a waiting list control group 18/28 Authors concluded that short-term CBT improves sleep in people with insomnia secondary to chronic pain.
Edinger et al. 2005 [41] USA, date not stated Clinic Parallel group RCT	Fibromyalgia (ACR diagnosis) aged 21–65 with insomnia 47 (18 CBT; 11 control; 18 sleep hygiene) Mean age 49 years 96% female	CBT for insomnia 6 weekly individual sessions. First 45–60 min, then 15–30 min Clinical psychologists Fidelity not reported Usual care Sleep hygiene		Baseline, end of treatment week 3, post-intervention, 6 months Insomnia symptoms questionnaire. McGill Pain questionnaire, no general HRQoL, POMS, SF-36 MH. Adverse events not reported. Overall loss to follow up 45% at 6 months Unclear risk of bias concern due to limited reporting. High risk at 6 months due to high loss to follow up and in comparisons including control due to uneven randomisation. Small study 19/28 The CBT group achieved nearly a 50% reduction in their nocturnal wake time by study completion compared with 3.5% in usual care and 20% in sleep hygiene groups. 57% of CBT group met strict subjective sleep improvement criteria at end of treatment compared with 0% in usual care and 17% in sleep hygiene groups.
Jungquist et al. 2010 [42] USA, dates not stated Community Parallel group RCT	Chronic spinal pain with insomnia. Age 25+ years 28 (19;9) Mean age 49 years 78% female	CBT for insomnia 8 weekly sessions (30–90 min) Trained masters prepared nurse therapist Fidelity assessed with sleep diaries and actigraphy Appointment with nurse therapist. Reviewed sleep/pain diaries and BDI items above a scale score of 0 for prior week. Discussed rationale that life stress and depression likely to contribute to insomnia and pain. No directed form of therapy provided. 8 weekly sessions, 45–90 min Nurse therapist		Baseline, post-treatment (8 weeks) Sleep diaries, actigraphy, insomnia severity index, Epworth sleepiness scale. Pain disability index, multidimensional pain inventory. No HRQoL or anxiety, BDI Adverse events collected on checklist of symptoms but not reported in article. Overall loss to follow up 25% Low risk of bias. 23/28 Significant improvements were found in sleep as well as in the extent to which pain interfered with daily functioning.
McCrae et al. 2019 [43] USA, 2009–2013 Clinic Parallel group RCT	Fibromyalgia (ACR criteria) and insomnia aged 18 years or older 113 (39; 37; 37) Mean age 53 years 97% female	CBT for insomnia. Sleep education, sleep hygiene and stimulus control, relaxation, sleep restriction, cognitive therapy – monitoring automatic thoughts, cognitive therapy – challenging/ replacing dysfunctional thoughts, cognitive therapy – practical recommendations, review of skills and long-term maintenance 8 weekly 50 min sessions Predoctoral students in clinical psychology with training and weekly supervision All treatment sessions audiotaped. Half were randomly selected for scoring by another interventionist, and 25% of the scored tapes were double-scored for reliability by the lead supervising clinical psychologist. Interventionists encouraged adherence and emphasised importance of regular home practice, which was monitored by daily practice logs CBT for pain. Pain education and diaphragmatic breathing, progressive muscle relaxation, activity-rest cycle and autogenic relaxation, visual imagery, cognitive therapy – monitoring automatic thoughts, cognitive therapy – challenging /replacing dysfunctional thoughts, cognitive therapy – balanced thinking, review of skills and long-term maintenance 8 weekly 50 min sessions Predoctoral students in clinical psychology with training and weekly supervision Fidelity as with CBT for insomnia Waiting list control		Baseline, post-treatment, 6 months Self-report sleep diary, dysfunctional beliefs and attitudes about sleep, actigraph, ambulatory polysomnography. McGill Pain questionnaire, VAS pain, Pain Disability Index. No HRQoL, STAI, BDI. Adverse events reported. Overall study loss to follow up 35% Unclear risk of bias due to concern for high loss to follow up 24/28 CBT for insomnia and CBT for pain led to improvements in self-reported sleep and this was sustained at 6 months after CBT for insomnia. No differences in pain compared with control group
Pigeon et al. 2012 [44] USA, date not reported Setting not reported Parallel group feasibility/ pilot RCT	Chronic non-malignant pain in the spine, shoulders, hips or limbs (unrelated to autoimmune disease or fibromyalgia) and insomnia 21 (6;6;5;4) Mean age 51 years 67% female	CBT for insomnia 10 individual weekly sessions 2 CBT psychologists Sessions videotaped and rated using treatment fidelity instrument created for study CBT for insomnia and pain 20 individual weekly sessions (1 each of CBT modalities) 2 CBT psychologists Sessions videotaped and rated using treatment fidelity instrument created for study CBT for pain 10 individual weekly sessions 2 CBT psychologists Sessions videotaped and rated using treatment fidelity instrument created for study Waiting list control		Baseline, 10 weeks (end of intervention) Insomnia severity scale, Epworth sleepiness scale, sleep diary. Multidimensional Pain Inventory, no HRQoL. Adverse events not reported No losses to follow up Unclear risk of bias for wait list controls. Pilot/ feasibility study 20/28 Authors concluded that a combined CBT for insomnia and pain intervention was feasible to deliver.
Smith et al. 2015 [45] USA, 2008–2013 Clinic Parallel group RCT	Knee osteoarthritis and insomnia 100 (50; 50) Mean age 59 years 79% female	CBT for insomnia including sleep restriction therapy, stimulus control therapy, cognitive therapy for insomnia, and sleep hygiene education. 8 × 45 minute weekly sessions Postdoctoral clinical psychologists, doctoral psychology candidates or faculty with experience in behavioural medicine. Structured checklist, supervision of intervention providers, sessions taped, and random sample assessed. Behavioral desensitization – manual adapted to match the CBT insomnia protocol for session number and duration. 8 × 45 minute weekly sessions Postdoctoral clinical psychologists, doctoral psychology candidates or faculty with experience in behavioural medicine. Structured checklist, supervision of intervention providers, sessions taped, and random sample assessed.		Baseline, mid-treatment, post-treatment, 3 months, 6 months Sleep diaries, polysomnography, actigraphy measures, Insomnia Severity Index. WOMAC pain (also VAS pain). No HRQoL or psychological health measures. No aAdverse events reported. Overall loss to follow up 27% Low risk of bias 22/28 In people with knee osteoarthritis, CBT for insomnia reduced sleep maintenance insomnia and clinical pain compared with active control.
Smitherman et al. 2016 [46] USA, 2011–2013 Clinic Parallel group RCT	Chronic migraine and ICSD-3 criteria for insomnia 32 (16; 16) Mean age 31 years 30.8 (12.9) 90% female	CBT for insomnia with 4 instructions in stimulus control and 1 in sleep restriction. 3 × 30 min sessions, with 2 weeks between each session. Graduate-level therapists with backgrounds in CBT and behavioural medicine Therapist fidelity to treatment protocols was assessed at each treatment session via therapist self-ratings Lifestyle modification with 5 skills taught and practiced at home including dinner at consistent time each night and consistent liquid intake. 3 × 30 min sessions, with 2 weeks between each session. Graduate-level therapists with backgrounds in CBT and behavioural medicine Therapist fidelity to treatment protocols was assessed at each treatment session via therapist self-ratings		Baseline, 2 and 6 weeks after completing treatment Actiwatch II, PSQI, Epworth Sleepiness Scale. Headache severity (0–10), no HRQoL, GAD-7 anxiety, PHQ-9 depression. Adverse events not reported. Overall loss to follow up 22% Low risk of bias, analysis was ITT 24/28 Significant group differences favoring CBT for insomnia in PSQI scores, total sleep time and sleep efficiency. No difference in Epworth Sleepiness Scale or change in headache severity between groups.
Tang et al. 2012 [47] UK, date not stated Clinic Parallel group pilot RCT	Chronic pain (non-malignant, not neurological) and clinical insomnia. Age 18–65 years 24 (12;12) Mean age 48.5 years 90% female	Hybrid of CBT for insomnia combined with interventions designed to target cognitive-behavioural processes maintaining chronic pain – sleep psychoeducation, stimulus control therapy, sleep restriction therapy, cognitive therapy, individual formulation, goal setting, reducing pain catastrophizing, reversing mental defeat 4 weekly 2 h individual sessions Clinician and health psychologist Intervention according to checklist which specified the content of each session. All sessions were video recorded. A sample of the recordings (20%) was independently reviewed for treatment fidelity. Wait list control. Monitoring group kept a pain and sleep diary for 4 weeks and then received the hybrid CBT intervention		Baseline, after treatment (4 weeks) Insomnia Severity Index, Anxiety and Preoccupation about sleep questionnaire, dysfunctional beliefs and attitudes about sleep questionnaire, pain-specific sleep beliefs, pre-sleep arousal scale, sleep onset latency, wake after sleep onset, total sleep time, sleep efficiency. Brief Pain Inventory. No HRQoL. HADS anxiety and depression. Adverse events not reported. Overall loss to follow up 17% Unclear risk of bias for wait list controls 19/28 Described as pilot RCT but authors reported that compared with symptom monitoring, the hybrid intervention was associated with greater improvement in sleep (as measured with the Insomnia Severity Index and sleep diary) at post-treatment.
Vitiello et al. 2009 [48] USA, 2001–2003 Academic medical centre Parallel group RCT	Osteoarthritis with moderate pain and insomnia. Age 55 or older 51 (23; 28) Mean age 69 years 88% female	CBT for insomnia. Stimulus control, sleep restriction, cognitive restructuring, relaxation training, sleep-hygiene education. 8 weekly 2 h classes Two clinical psychologists Fidelity not reported Attention-control stress management and wellness. Skill-training on mind-body relationship, reduction of stress and anxiety, effective communication and assertiveness, problem solving and goal setting, nutrition and exercise for individuals with chronic conditions. 8 weekly 2 h classes Physician, psychologist, nutritionist, exercise physiologist		Baseline (before treatment), post-treatment. One year not considered as included cross-over patients. Total sleep time, naps, sleep latency, wake after sleep onset, sleep efficiency. SF-36 bodily pain, no HRQoL measure, GDS. Adverse events not reported Losses to follow up not reported Unclear risk of bias due to limited reporting 21/28 CBT for insomnia improved immediate self-reported sleep and pain in older patients with osteoarthritis and comorbid insomnia compared with an attention control.
**1b. Brief cognitive behavioural therapy and sleep hygiene compared with wait list controls**				
Berry et al. 2015 [49] Canada, dates not stated Outpatient clinic Parallel group RCT	Chronic non-cancer pain including neuropathic pain, musculoskeletal pain, complex regional pain syndrome, joint pain, visceral pain and headaches, age 18–80 years 132 (65;67) Mean 49 years 61% female	Education with CBT component. Education session incorporating sleep hygiene and cognitive behavioral strategies 15 min session once. Subsequently participants contacted weekly for 4 weeks by telephone to address questions or concerns about completing diaries and to ensure they were engaging in treatment strategies. Trained research assistant Consistency of intervention delivery monitored No treatment but offered after study completion		Baseline, week 1, week 2, week 3 during intervention. Week 4 at end of intervention Daily sleep diary. No pain, HRQoL or psychological measures. Adverse events not reported Overall 36% lost to follow up 20/28 High risk of bias due to large loss to follow up No differences between groups except sleep latency improved in Education group
**1c. Cognitive behavioral therapy focusing on insomnia compared with sleep hygiene**				
Edinger et al. 2005 [41] Details above				
Martinez et al. 2014 [50] Spain, dates not reported Clinic Parallel group RCT	Women with fibromyalgia, aged 25–60 years 64 (32;32) Mean age 48 years 100% female	CBT for insomnia 6 group 1.5 h sessions, 1 per week. 5–6 participants per group. Female therapists with experience of pain management and sleep disorders. Fidelity not reported Sleep hygiene 6 group 1.5 h sessions, 1 per week. 5–6 participants per group. Female therapists with experience of pain management and sleep disorders. Fidelity not reported		Baseline, post-treatment, 3 months, 6 months PSQI. McGill pain questionnaire (Spanish), FIQ, SCL-90-R. Adverse events not reported Overall loss to follow up 27% Low risk of bias 19/28 Authors reported that patients who received CBT for insomnia reported significant, positive and sustained changes in subjective sleep quality, sleep latency, sleep duration, habitual sleep efficiency and sleep disturbances. Patients in the sleep hygiene group only reported a significant improvement in subjective sleep quality and a trend towards improvement in sleep efficiency.
Miro et al. 2011 [51] Spain, dates not reported Group Parallel group RCT	Women with fibromyalgia and insomnia 44 (22;22) Mean age 46 years 100% female	CBT for insomnia 6 weekly group sessions lasting 90 mins Female CBT experts with experience in fibromyalgia Fidelity not reported Sleep hygiene 6 weekly group sessions lasting 90 mins Female CBT experts with experience in fibromyalgia Fidelity not reported		Baseline, 1 week post-intervention PSQI. McGill pain questionnaire (Spanish). Adverse events not reported Overall loss to follow up 9% Low risk of bias 23/28 Authors reported that compared with the sleep hygiene group, patients receiving CBT for insomnia had improved sleep quality. No difference in pain between groups
Sanchez et al. 2012 [52] Spain, dates not reported Clinic Parallel group RCT	Fibromyalgia with chronic insomnia. Age 25–60 years 26 (13;13) Mean age 47 years 100% female	CBT for insomnia 6 weekly group sessions of 90 mins each Female CBT experts with experience in fibromyalgia Fidelity not reported Sleep hygiene 6 weekly group sessions of 90 mins each Female CBT experts with experience in fibromyalgia Fidelity not reported		Baseline, 6 weeks (post-treatment) Polysomnographic parameters. No pain outcome. Adverse events not reported. Losses to follow up not reported Low risk of bias 22/28 Authors conclude that use of CBT for insomnia in fibromyalgia patients can significantly improve objective sleep parameters
**1d. Cognitive behavioural therapy focusing on insomnia and pain compared with control**				
Castel et al. 2012 [53] Spain, dates not stated Clinic Parallel group RCT	Fibromyalgia, age 18–65 years 93 Mean age 50 years 97% female	CBT for pain and insomnia 14 weekly sessions, 120 mins each, all group except session 2 Not described who delivered No information on fidelity CBT for pain and insomnia plus hypnosis 14 weekly sessions 120 mins each, group except session 2. Session 2 received hypnosis training. Hypnosis exercises performed at end of each session instead of autogenic. Audio compact disc for home practice with analgesic self-hypnosis exercises. Not described who delivered. No information on fidelity Standard care		Baseline, 1 week post-treatment, 3 months, 6 months Medical outcomes study sleep scale. NRS pain, FIQ, HADS total. Adverse events not reported Overall loss to follow up 24% Unclear risk of bias due to limited reporting 17/28 CBT for pain and insomnia showed greater improvements in sleep than standard care. Adding hypnosis led to increased benefit.
Lami et al. 2018 [54] Spain, dates not reported Clinic, group Parallel group RCT	Women with fibromyalgia and insomnia aged 25–65 years 126 (42;42;42) Mean age 50 years 100% female	CBT for insomnia and pain 9 weekly 90 min group (5–7 people) sessions Therapists with high level professional training and experience in chronic pain and sleep disorders Fidelity assessed with video recordings and regular meetings CBT for pain 9 weekly 90 min group (5–7 people) sessions Therapists with high level professional training and experience in chronic pain and sleep disorders Fidelity assessed with video recordings and regular meetings Control group receiving usual care		Baseline (pre-treatment), 1 week after completion of the intervention, 3 months post-treatment PSQI. McGill Pain Questionnaire Short Form, FIQ, SCL-90-R. Adverse events not reported Large losses to follow-up (28%) at post-treatment timepoint High risk of bias due to large loss to follow up 20/28 Authors report significant improvement in sleep variables in CBT for pain and insomnia compared with CBT for pain and control. CBT groups had improvements in pain outcomes compared with control
Pigeon et al. 2012 [44] Details above				
Vitiello et al. 2013 [55] USA, 2009–2011 Classroom or primary care clinic Cluster RCT	Osteoarthritis and insomnia aged 60+ 367 (122;122;123) Mean age 73 years 75% female	CBT for pain and insomnia 6 weekly 90-min sessions 2 female mental health professionals (MSc level family counselor and PhD psychologist) Authors report ongoing treatment fidelity monitoring CBT for pain 6 weekly 90-min sessions 2 female mental health professionals (MSc level family counselor and PhD psychologist) Authors report ongoing treatment fidelity monitoring Education only control 6 weekly 90-min sessions 2 female mental health professionals (MSc level family counselor and PhD psychologist) Authors report ongoing treatment fidelity monitoring		Baseline, 2 months (post-treatment), 9 months after baseline, 18 months Insomnia severity index, sleep efficiency via actigraphy. Graded Chronic pain Scale, no specific HRQoL (AIMS2 symptom subscale reflects pain). Adverse events not reported Overall loss to follow up 13% Low risk of bias 24/28 At 9 months CBT for pain and insomnia reduced insomnia severity compared with CBT for pain and control. No significant differences between CBT groups and control in sleep or pain outcomes at 18 months.
***1e. Cognitive behavioural therapy focusing on insomnia and pain*versus*cognitive behavioural therapy for pain***				
Lami et al. 2018 [54] Details above				
McCrae et al. 2019 [43] Details above				
Pigeon et al. 2012 [44] Details above				
Vitiello et al. 2013 [55] Details above				
***1 f. Cognitive behavioural therapy focusing on insomnia and pain alone or with additional hypnosis***				
Castel et al. 2012 [53] Details above				
***1 g. Acceptance and Commitment Therapy based stress management compared with control*** ***1 h. Acceptance and Commitment Therapy based stress management compared with exercise***				
Wiklund et al. 2018 [56] Sweden, dates not reported Clinic/conference room at hospital/ training facility Parallel group RCT	Adults (18–60 years) with chronic (> 3 months) benign neck, low back, and/or generalised pain 299 (99 ACT;100 control; 100 Exercise) Mean age 54 years % female not reported	Acceptance and commitment therapy (ACT) based stress management 7 weekly 2-h sessions Specialist ACT psychologists 61% completed 4 or more sessions Group-based exercise. Graded exercises: endurance, coordination, balance, functional strength, and movement training. After 4 weeks: increased aerobic training and decreased range of movement exercises. Individualised exercise: graded strength exercises for back, neck, abdomen, shoulders, and arms. Home exercise. One hour, twice a week for 8 weeks. Physiotherapist and physician. 65% completed 9 or more sessions Control group with group discussion of persistent pain and participants’ experiences moderated by medical or psychology student, research nurse, or licensed psychologist. 7 weekly 2-h meetings. 60% completed 4 or more sessions		Baseline, immediately post-intervention,6 and 12 months Insomnia Severity Index. Pain NRS for average pain intensity over past 7 days, no HRQoL measure, HADS. Adverse events not reported. Overall loss to follow up 25% at 6 months 20/28 Unclear risk of bias due to selective reporting. Unclear risk of bias due to losses to follow up at 6 months No benefit for ACT-based stress management over control or exercise therapy
***1i. Mindfulness compared with control***				
Cash et al. 2015 [57] USA, date not specified Clinic, group Parallel group RCT	Women volunteers aged 18+ with physician diagnosed fibromyalgia 91 (51;40) Mean age not reported 100% female	Mindfulness-based stress reduction group. Formal and informal mindfulness practices including attention-focusing, sitting meditation and yoga positions taught to encourage relaxed and focused movement. Home practice guided by a workbook and audiotapes. Half-day meditation retreat Experienced, trained instructor 1 weekly 2.5-h group session for 8 weeks. Home 45 min per day, 6 days a week No fidelity relating to delivery reported Waiting list controls		Baseline, after 8 week programme, 2 months after completion of programme Stanford Sleep Questionnaire. VAS pain. Adverse events not reported Overall loss to follow up 25% Unclear risk of bias, authors reported ITT with analysis including baseline data for losses to follow up. Wait list controls 20/28 Authors reported that the mindfulness intervention was associated with significant and maintained reductions of sleep problems and symptom severity compared with controls
Van Gordon et al. 2017 [58] UK, 2012–2014 Community Parallel group RCT	Fibromyalgia. Age 18–65 years 148 (74;74) Mean age 47 years 83% female	Meditation awareness training – Second-generation mindfulness-based intervention. Taught component; facilitated group discussion, guided meditation and/or mindfulness exercises. One-to-one support sessions and compact disc of guided meditations to facilitate daily self-practice. 8 weekly 2 h 25 participant workshops plus CD of guided meditation for self-practice. 2 one-to-one support sessions. Instructors attended a 3-year supervised training programme. At least 15 min of each weekly session were observed and discussions held with the programme facilitator on a weekly basis. Group CBT with education focus. Taught presentation, facilitated group discussion component, guided discovery educational exercises with the same number and duration of breaks as the target intervention. Identical to the intervention condition on all non-specific factors such as overall course length, individual session duration, group and one-to-one discussion component, group size, and inclusion of an at-home practice element Programme duration and intensity, deliverer, and fidelity assessment as with Meditation awareness training intervention.		Baseline, post-treatment, 6 month follow-up PSQI. Short Form-McGill Pain Questionnaire, DASS. Adverse events not reported Losses to follow-up at final assessment 43% High risk of bias due to large loss to follow up and retrospective registration 24/28 Authors report that Meditation awareness training participants demonstrated significant and sustained improvements over control group participants in sleep quality and pain perception.
***1j. Relaxation compared with control***				
Soares and Grossi 2002 [59] Sweden, dates not reported Community Parallel group RCT	Women with fibromyalgia 53 (18;18;17) Mean age 45 years 100% female	Behavioural intervention. Individual sessions focused on applied relaxation. Group sessions covering symptoms, stress, behaviour patterns and self-management 5 individual sessions (1 h each) and 15 group sessions (2 h each:3–5 patients in each group) over 10-weeks Experienced CBT therapist and pain physician Sessions observed Educational intervention with a sleep hygiene focus 2 individual sessions (2 h each) and 15 group sessions (2 h each/3–5 patients per group) over 10 weeks Experienced physiotherapist and occupational therapist Sessions observed Waiting list control		Baseline (before treatment), after treatment and 6 months (only post-treatment for wait list controls) Karolinska Sleep Questionnaire.McGill Pain Questionnaire, The Pain Questionnaire, pain VAS, FIQ. No psychological measures. Adverse events not reported No losses to follow up post-treatment. Overall 23% lost to follow up at 6 months Unclear risk of bias for relaxation/ education comparison due to wait list control 18/28 Behavioural intervention associated with long-term improvement in sleep quality. No other differences between groups at long-term follow up
**2. Sleep hygiene compared with control**				
Edinger et al. 2005 [41] Details above				
Soares and Grossi 2002 [59] Details above				
**3. Exercise**				
**3a. Group exercise compared with control**				
Arcos-Carmona et al. 2011 [60] Spain, 2008–2009 Clinic Parallel group RCT	Fibromyalgia (ACR diagnosis) in previous 2 years, age 30–60 years 56 (28; 28) Mean age 44 years 100% female	Aerobic exercise plus relaxation. 30 min graded aerobic exercise in pool followed by 30 mins progressive relaxation Twice a week for 10 weeks Not stated who delivered Fidelity not reported Sham magnetotherapy. Patients lying down with the screen covered to avoid detecting that the machine was disconnected. 10 min cervical spine, 10 min lumbar. Twice a week for 10 weeks Not stated who delivered Fidelity not reported		Baseline, at end of 10 week intervention PSQI. SF36 bodily pain. Adverse events not reported Overall 5% lost to follow up Unclear risk of bias due to limited methodological reporting 20/28 The authors report that the combination of aerobic exercise and progressive relaxation improved night rest
Durcanet al. 2014 [61] Ireland, date not stated Home Parallel group RCT	Rheumatoid arthritis 80 (42;38) Mean age 60 years 64% female	Home-based cardiovascular exercise, resistance training, flexibility and neuromotor conditioning. 12 weeks Assessment by doctor and senior physiotherapist every 3 weeks Fidelity not described Advice on benefits of exercise		Baseline and post-treatment (12 weeks) PSQI. VAS pain. Adverse events not reported Overall loss to follow up 3% Low risk of bias 23/28 Significant improvement in sleep quality and pain in exercise group but not in control group
Eadie et al. 2013 [62] Ireland, 2008–2010 Outpatient Parallel group feasibility RCT	Chronic or recurrent non-specific low back pain. Age 18–70 years 60 (20 Home-based walking; 20 Control; 20 Supervised exercise) Mean age 45 years 62% female	Home-based walking programme 8 weeks. 30 min moderate-intensity physical activity, 5 days/ week by week 5 Physiotherapist 77.5% patient adherence Supervised exercise class Once per week for 8 weeks Physiotherapist 50% patient adherence Control of usual physiotherapy. 1-to-1 advice, manual therapy and exercise Number and duration at discretion of treating physiotherapist Physiotherapist Fidelity not described (Supervised exercise class)		Baseline, 3 and 6 months (equivalent to post-treatment and 3 months) PSQI, Insomnia Severity Index, Pittsburgh Sleep Diary, actigraphy. NRS pain, SF-36 MH, HADS. Adverse events documented reported to the trial coordinator Overall 30% lost to follow up at 6 months Unclear risk of bias. High risk of bias at 6 months follow up due to high loss to follow up 21/28 Feasibility study only
Freburger et al. 2010 [63] USA, dates not stated Community Parallel group RCT	Self-report arthritis and activity limitations, 18 years or over 321 (166; 155) Mean age 70 years 88% female	Exercise programme. Low-to-moderate-intensity physical activity program: People With Arthritis Can Exercise (PACE)/ Arthritis Foundation Exercise Program. Land-based exercise, health education on arthritis self-management and exercise. Activities to promote social interaction, movement, balance, and body awareness. Relaxation techniques. I hour, twice weekly for 8 weeks PACE trained instructor Fidelity not assessed Waiting list control		Baseline, 8 weeks Jenkins sleep scale. Pain not reported. Adverse events not reported Overall 15% lost to follow up Unclear risk of bias due to wait list control 21/28 No sustained benefit for improved sleep beyond the 8 week, end of programme assessment
Wiklund et al. 2018 [56] Details above				
**3b. Home-based walking programme versus supervised exercise**				
Eadie et al. 2013 [62] Details above				
**3c. Moderate aerobic exercise compared with low intensity home-based exercise**				
Al-Sharman et al. 2019 [64] Jordan, 2015–2018 Clinic and control at home Parallel group pilot RCT	Multiple sclerosis with poor sleep quality, age > 18 years 40 (20;20) Mean age 35 years 77% female	Moderate-intensity aerobic exercise programme. Supervised moderate-intensity aerobic exercise using a recumbent stepper machine. Upper and lower body stretching exercises before and after each exercise session 40 min per session, 3 times a week for 6 weeks Not stated who delivered Weekly exercise logs Low-intensity home-exercise programme. DVD and exercise manual. DVD showed warm up and cool down activities, flexibility, strength, balance and endurance exercises. Also, relaxation, stretching, and breathing techniques. 50–60 min per session, 3 sessions a week for 6 weeks Participants asked to demonstrate exercises with examiner. Weekly exercise logs		Baseline, end of intervention (6 weeks) PSQI, Insomnia Severity Index and Actigraph in subsample of patients. Pain not reported. Adverse events not reported Overall loss to follow up 25% High risk of bias due to high and uneven loss to follow up and limited reporting of methods 19/28 Pilot study only
**3d. Aquatic biodance compared with stretching exercises**				
Lopez-Rodriguez et al. 2013 [65] Spain, 2011 Clinic Parallel group RCT	Fibromyalgia (ACR diagnosis) in previous 2 years, age 18–68 years. 76 (38;38) Mean age 55 years 100% female	Aquatic biodance for 1 h in pool heated to 29C. 10 min flexibility/ breathing exercises, 40 min creative dance movement to music involving upper/lower limbs, 10 min gentle exercise Twice weekly 1 h session for 12 weeks Not stated who delivered Fidelity not reported Stretching exercises including neck, trunk, quadriceps and calves. Twice weekly 1 h sessions for 12 weeks		Baseline, post-treatment PSQI. McGill Pain questionnaire, FIQ, STAI, CES-D. Adverse events not reported Overall 22% lost to follow up Unclear risk of bias, concern for loss to follow up but ITT 22/28 Intervention associated with improvements in sleep quality and pain
***3e. Tai Ji Quan***				
Lu et al. 2017 [66] China, 2013 Community Parallel group RCT	Knee osteoarthritis aged 60–70 years 46 (23;23) Mean age 65 years 100% female	Tai Ji Quan: 8 forms adapted for use in people with osteoarthritis 60 min session 3 times weekly for 24 weeks Two instructors with training and academic specialisation in Tai Ji Quan Specialist monitored fidelity of delivery on a weekly basis Education focusing on wellness and health promotion 60 min class twice per week for 24 weeks Additional 10–15 min weekly check-in phone call from research staff to monitor activity levels, changes in knee pain, and medication usage Fidelity not reported		Baseline (before intervention), at end of study (after intervention), 24 weeks PSQI. WOMAC pain, SF-36 MH. Adverse events not reported Overall 13% lost to follow up at 24 weeks Low risk of bias 23/28 Significant improvement in sleep measures and pain in Tai Ji Quan group compared with controls
Maddali Bongi et al. 2016 [67] Italy, dates not stated Community and home Parallel group RCT	Women with fibromyalgia (ACR criteria) 44 (22;22) Mean age 52 years 100% female	Tai Ji Quan: breathing exercises, concentration, postural maintenance, rebalancing, and precise movement. Twice weekly 60 min sessions for 16 weeks. Daily DVD led home exercises in two 15 min sessions Not reported who delivered Fidelity not described Education about the disease, symptoms, management and coping Twice weekly 60 min sessions for 16 weeks Fidelity not described		Baseline, 16 weeks (end of treatment) PSQI. Widespread pain index, HADS. Adverse events not reported No losses to follow up reported (6 withdrew before study) Unclear risk of bias due to limited reporting 20/28 Improvement in some sleep parameters and widespread pain in Tai Ji Quan group but not in education group
**4. Physical therapy**				
**4.1. Hydrotherapy**				
Calandre et al. 2009 [68] Spain, dates not stated Clinic Parallel group RCT	Fibromyalgia aged 18+ years 81 (39;42) Mean age 50 years 90% female	Hydrotherapy exercise with stretching 18 sessions of 1 h, 3 times per week for 6 weeks Physiotherapist Fidelity not reported Hydrotherapy exercise with Tai Chi 18 sessions of 1 h, 3 times per week for 6 weeks Physiotherapist Fidelity not reported		Baseline, end of treatment (6 weeks), 4 weeks after end of treatment, 12 weeks after end of treatment PSQI. VAS pain, FIQ, STAI, Bdi. Adverse reactions listed Overall loss to follow up 30% High risk of bias, large loss to follow up 19/28 No differences were found between groups
Vitorino et al. 2006 [69] Brazil, dates not reported Clinic Parallel group RCT	Women with fibromyalgia 50 (25;25) Mean age 48 years 100% female	Hydrotherapy with warm-up, stretching, aerobic exercises and relaxation 60 mins, 3 times per week for 3 weeks Physiotherapist Fidelity not described Conventional physiotherapy with infra-red lamp, stretching, aerobic exercise and relaxation 60 mins, 3 times per week for 3 weeks Physiotherapist Fidelity not described		Baseline (pre-treatment), post-treatment. Sleep logs completed for 21 days before and after treatment Total sleep time, total nap time. SF-36 bodily pain, SF-36 MH. Adverse events not reported Overall loss to follow up 6% Low risk of bias 21/28 Sleep quality showed grater improvement in hydrotherapy group but no difference in improvement in pain outcome
**4.2. Massage or Manual therapy compared with relaxation or control**				
Field et al. 2007 [70] USA, dates not stated Group clinic and home-based Parallel group RCT	Chronic lower back pain 30 total Mean age 41 years 47% female	Massage therapy 30 mins twice per week for 5 weeks Trained massage therapists Fidelity not reported Relaxation therapy 30 mins twice per week for 5 weeks Home-based Patient log		Baseline, 5 weeks (post-treatment) Verran and Snyder-Halperin Sleep scale. VAS pain, POMS, STAI. Adverse events not reported Losses to follow up not reported Unclear risk of bias for limited reporting. Baseline difference in sleep disturbance 20/28 Authors report sleep disturbance and pain less in massage therapy group
Castro-Sanchez et al. 2014 [71] Spain, dates not stated Clinic Parallel group RCT	Fibromyalgia, age 18–70 years 89 (45;44) Mean age 54 years 53% female	Manual therapy 5 weekly sessions of 45 mins each Specialist physiotherapist Fidelity not described No treatment		Baseline, post-intervention (48 h after end of 5 week intervention) PSQI. McGill Pain Questionnaire. Adverse events not reported No losses to follow up Unclear risk of bias due to lack of blinding 23/28 Authors concluded that manual therapy was effective in improving sleep and pain
**4.3. Physical therapy programme**				
Külcü et al. 2009 [72] Turkey, 2006–2007 Rehabilitation clinic Parallel group RCT	Primary fibromyalgia age 18–55 years 60 (40;20) Mean age 37 year 95% female	Physical therapy programme with hot pack, ultrasound, TENS and low power laser 15 sessions Fidelity not described No physical therapy		Baseline and at end of intervention Insomnia Severity Index. VAS pain. Adverse events not reported No losses to follow up High risk of bias due to limited reporting and lack of blinding 19/28 Sleep and pain improved in physical therapy group compared with controls
**4.4. Pompage and stretching and aerobic exercise compared with stretching and aerobic exercise**				
Correia Moretti et al. 2016 [73] Brazil, 2011–2013 Clinic Parallel group RCT	Fibromyalgia, aged 18–60 years 23 (13;10) Mean age 45 years 100% female	Pompage. Global, lymph, trapezius, torso, lumbar, and quad pompage plus stretching and aerobic exercise Twice per week for 12 weeks Not specified who delivered Fidelity not reported Stretching and aerobic exercise Twice per week for 12 weeks Not specified who delivered Fidelity not reported		Baseline, 6 weeks, 12 weeks (post-treatment) Sleep inventory. McGill Pain Questionnaire, no HRQoL or psychological health measures. Adverse events not reported Overall 35% lost to follow up High risk of bias for high and uneven loss to follow up 22/28 Authors report no benefit for pompage for sleep and limited benefit regarding pain
**5. Miscellaneous interventions**				
**5.1. Acupressure**				
Yeh et al. 2016 [74] USA, dates not reported Clinic/ office Parallel group RCT	Chronic low back pain 61 (30;31) Mean age 63 years 67% female	Auricular point acupressure. Vaccaria seeds placed on active ear points corresponding to low back pain and alleviation of stress and pain. Participants told to press the seeds on each ear at least 3 times a day for 3 min and whenever they experienced pain. Seed removed after 5 days Four weekly clinic visits to place seeds Not specified who delivered Participants completed treatment diaries Sham auricular point acupressure. Vaccaria seeds taped to stomach, mouth, duodenum, and eye acupoints of ears Four weekly clinic visits to place seeds Not specified who delivered Participants completed treatment diaries		Baseline, during each of the 4 treatments, end of intervention, and 1 month after the last treatment PSQI, daily sleep diary. BPI short form, no HRQoL or psychological health measures. Adverse events not reported Overall 25% lost to follow up High risk of bias for high and uneven loss to follow up 20/28 Authors reported that auricular point acupressure led to improvement in several sleep parameters and pain
Murphy et al. 2019 [75] USA, 2013–2016 Clinic Parallel group RCT	Chronic nonspecific low back pain and fatigue. Aged 18+ years 67 (22;22;23) Mean age 50 years 63% female	Self-administered relaxing acupressure at 9 points. Pressure applied to each point in circular motion 27–30 min per day for 6 weeks, each point for 3 min Trained acupressure educators Standardised training of educators, proper enactment of intervention and methods to track adherence Self-administered stimulating acupressure at 10 points. Pressure applied to each point in circular motion 27–30 min per day for 6 weeks, each point for 3 min Trained acupressure educators Standardised training of educators, proper enactment of intervention and methods to track adherence Usual care. Weekly calls regarding health status to ensure similar levels of researcher contact		Baseline, post-treatment (6 weeks) PSQI. Brief Pain Inventory. Adverse events reported Overall loss to follow up 18% Unclear risk of bias due to limited reporting 23/28 Authors report no improvement in sleep quality between acupressure groups and compared with control. Pain reduced in acupressure groups but no change in control
**5.2. Bright light therapy**				
Pearl et al. 1996 [76] Canada, 1992–1993 Home Crossover RCT	Fibromyalgia. Aged 21–65 years 19 randomised but crossover protocol completed by 14 people Mean age 38 years 100% female	Bright light therapy delivered by a visor with Krypton incandescent bulbs with a mean of 4750 (SD 2337) lux 4 weeks of 1 condition, break week, and 4 weeks alternative treatment Administered at home Fidelity not reported No light condition. Visor system fitted with opaque filter (exposed Kodak film) 4 weeks of 1 condition, break week, and 4 weeks alternative treatment Administered at home Fidelity not reported		Pre-light, light week 4, pre no light, no light week 4 Daily post sleep questionnaire, VAS sleep quality, daily sleep diary. Daily NRS for pain in 10 body regions, FIQ anxiety and depression. Adverse events not reported Overall 26% did not complete crossover protocol High risk of bias due to large loss to follow up and limited reporting of methods 21/28 Authors reported no significant differences between light and no light conditions on sleep or pain
**5.3. Foot reflexology**				
Bakir et al. 2018 [77] Turkey, 2015 Clinic Parallel group RCT	Rheumatoid arthritis aged 18+ years 68 (34;34) Mean age 50 years 77% female	Foot reflexology 60 min repeated once a week for 6 weeks Certified researcher Treatment fidelity not reported Routine polyclinic monitoring and information		Baseline, 1 week, 6 weeks (pain recorded weekly) PSQI. VAS pain. No HRQoL or psychological health. Adverse events not reported 12% lost to follow up Unclear risk of binding due to lack of blinding 20/28 Authors report that sleep and pain improved in the foot reflexology group
**5.4. Transcranial stimulation**				
Harvey et al. 2017 [78] Canada, dates not stated Laboratory Parallel group feasibility RCT	Stable musculoskeletal pain and insomnia, age 60+ years 16 (8;8) Mean age 71 years 81% female	Transcranial direct current stimulation (tDCS) applied over the primary motor cortex (2 mA, 20 min) 5 daily sessions of 20 min given in afternoon or evening Investigator No information on treatment fidelity Sham transcranial direct current stimulation applied over the primary motor cortex 5 daily sessions of 20 min given in afternoon or evening Investigator No information on treatment fidelity		Baseline, day 12 (post-treatment), and day 19 (7 days post-treatment). PSQI. Pain and sleep log books completed each day at home, actigraph days 1–19 (only available for 4 participants). VAS pain. No HRQoL or psychological health. Adverse events not reported 13% lost to follow up Feasibility study, unclear risk of bias due to limited reporting 21/28 Study provides guidelines for future studies. Authors report no difference in sleep parameters between groups
**5.5. Mattress**				
Colbert et al. 1999 [79] USA, 1997 Home Parallel group RCT	Fibromyalgia (ACR criteria) 30 (15; 15) Mean age 50 years 100% female	Magnetic mattress pad strength 1100 G which delivered 200–600 G to the skin surface. Delivered to patients with instructions on placement. Each night for 16 weeks. No information on fidelity Sham mattress pad, Delivered to patients with instructions on placement. Each night for 16 weeks. No information on fidelity		Baseline, 16 weeks VAS (sleep, fatigue, tiredness on waking, total sleep time). Pain VAS, FIQ-ADL, no psychological measure. Diary of adverse reactions. Low risk of bias Overall 17% lost to follow up. 23/28 Patients sleeping on the intervention magnetic mattress pad had improvement in reported sleep (*p* < 0.01) and decrease in pain (*p* < 0.05) with no adverse events related to the magnetic mattress pad.
Minetto et al. 2018 [80] Italy, dates not reported Home Parallel group pilot RCT	Chronic lower back pain 38 (28; 10) Median 58 years 58% female Both groups had usual rehabilitation exercises to improve back strength, balance and mobility. 1 h daily, 3 days per week for 2 months	Mattress overlay. Aiartex overlay with suspensory monofilaments to support a person lying in bed. No supervision. Each night for 2 months. All patients “compliant with its use” Usual rehabilitation only		Baseline, end of intervention at 2 months PSQI. VAS pain. No HRQoL or psychological health. Adverse events not reported. Losses to follow up not reported High risk of bias due to randomization procedure, non-blinding and reporting. 18/28 Authors report that the mattress overlay was associated with better and clinically meaningful sleep and pain compared with controls.

*ACR* American College of Rheumatology, *ACT* Acceptance and Commitment Therapy, *AIMS2* Arthritis Impact Measurement Scale 2, *BDI* Beck Depression Inventory, *BPI* Brief Pain Inventory, *CBT* Cognitive Behavioural Therapy, *CES-D* Center for Epidemiologic Studies Depression Scale, *DASS* Depression Anxiety Stress Scales, *FIQ* Fibromyalgia Impact Questionnaire, *GAD* General Anxiety Disorder-7, *GDS* Geriatric Depression Scale, *HADS* Hospital Anxiety and Depression Scale, *HRQoL* Health Related Quality of Life, *ITT* Intention To Treat, *NRS* Numeric Rating Scale, *PHQ-9* Patient Health Questionnaire, *POMS* Profile of Mood Sates, *PSQI* Pittsburgh Sleep Quality Index, *RCT* Randomised controlled trial, *SCL-90-R* Symptom Checklist-90-Revised, *SF-36 36-Item* Short Form Survey (SF-36), *SF-36-MH 36-Item* Short Form Survey (SF-36) Mental Health, *STAI* State-Trait Anxiety Inventory, *TENS* Transcutaneous Electrical Nerve Stimulation, *VAS* Visual Analogue Scale, *WOMAC* Western Ontario and McMaster Universities Arthritis Index

^a^Downs and Black Score modification as described in Hooper P, Jutai JW, Strong G, Russell-Minda E. Age-related macular degeneration and low-vision rehabilitation: a systematic review. Can J Ophthalmol. 2008 Apr;43(2):180–7. doi: 10.3129/i08-001. Quality levels: excellent (26–28); good (20–25); fair (15–19); poor (≤14)

The primary areas addressed by interventions were psychological, physical exercise, physical therapy, and other. Within these groups, comparisons were with untreated controls or alternate active interventions. Sleep outcomes were questionnaires focusing on specific aspects of sleep experience, sleep diaries including aspects of time in bed, nocturnal sleep time, sleep latency, sleep efficiency, wake after sleep onset, total sleep time, and number of awakenings, or measurements with sensors such as actigraphy or polysomnography. Pain outcomes were reported in 38 studies, health-related quality of life outcomes in 24 studies and measures of psychological health in 26 studies. Adverse events were infrequently recorded. All studies reported follow up at the end of intervention or within 2 weeks of completion. Fifteen studies reported follow up at 3 months or longer.

For all studies, effect estimates comparing intervention with control or alternative intervention are summarised in Supplement Table 5 with outcomes reported in multiple studies shown in meta-analysis summaries in Table 2.

**Table 2:** Effect estimates for interventions with multiple studies reporting outcomes

Follow up	Effect measure	Number of studies (participants)	Effect estimate (95%CI), ***p***-value	I^**2**^
**Cognitive behavioural therapy for insomnia versus control**				
***Sleep quality from questionnaire***				
Post-treatment	SMD	9 (203:183)	-1.23 (−1.76, − 0.70), *p* < 0.00001	80%
3 months	SMD	2 (65:68)	−0.80 (−2.17, 0.57), *p* = 0.25	93%
6 months	SMD	3 (64:70)	−0.51 (−1.02, − 0.01), *p* = 0.04	41%
***Sleep - onset latency (diary)***				
Post-treatment	MD	7 (168:160)	−17.42 (−28.41, −6.43), *p* = 0.002	73%
3 months	MD	2 (64:70)	−14.58 (−21.67, −7.50), < 0.0001	0%
6 months	MD	3 (62:68)	−6.48 (−14.36, 1.40), *p* = 0.11	0%
***Sleep - efficiency (diary)***				
Post-treatment	MD	8 (174:164)	−9.86 (−14.06, −5.66), *p* = < 0.00001	67%
3 months	MD	2 (64:70)	−9.36 (− 15.10, − 3.61), *p* = 0.001	53%
6 months	MD	3 (62:68)	−6.16 (−10.31, −2.01), *p* = 0.004	46%
***Sleep – wake after sleep onset (diary)***				
Post-treatment	MD	7 (168:160)	−31.12 (−43.55, − 18.69), *p* < 0.00001	71%
3 months	MD	2 (64:70)	−29.15 (−55.10, − 3.19), *p* = 0.03	67%
6 months	MD	4 (85:96)	−18.58 (−30.57, −6.58), *p* = 0.002	51%
***Sleep awakenings (diary)***				
Post-treatment	MD	2 (51:37)	−0.23 (− 0.88, 0.42), *p* = 0.48	0%
***Sleep - total sleep time (diary)***				
Post-treatment	MD	8 (174:164)	−6.70 (−31.98, 18.58), *p* = 0.60	58%
3 months	MD	2 (64:70)	−22.10 (−69.11, 24.91), *p* = 0.36	73%
6 months	MD	4 (85:96)	−2.44 (− 22.15, 17.26), *p* = 0.81	0%
***Sleep - onset latency (actigraph)***				
Post-treatment	MD	3 (80:81)	−3.89 (−14.55, 6.77), *p* = 0.47	63%
6 months	MD	3 (56:64)	−4.15 (−9.97, 1.68), *p* = 0.16	0%
***Sleep - efficiency (actigraph)***				
Post-treatment	MD	4 (96:96)	−1.58 (−5.59, 2.42), *p* = 0.44	50%
6 months	MD	3 (55:65)	−2.17 (−5.42, 1.09), *p* = 0.19	0%
***Sleep – wake after sleep onset (actigraph)***				
Post-treatment	MD	3 (80:81)	−13.12 (− 24.39, − 1.85), *p* = 0.02	0%
6 months	MD	3 (56:64)	−8.31 (−20.16, 3.53), *p* = 0.17	0%
***Sleep - Total sleep time (actigraph)***				
Post-treatment	MD	4 (96:96)	13.66 (−18.58, 45.91), *p* = 0.41	51%
6 months	MD	3 (56:64)	17.50 (−9.93, 44.93), *p* = 0.21	11%
***Sleep - onset latency (polysomnograph)***				
Post-treatment	MD	2 (65:74)	5.89 (−9.16, 20.93), *p* = 0.44	0%
6 months	MD	2 (56:59)	−5.24 (−23.33, 12.84), *p* = 0.57	0%
***Sleep - efficiency (polysomnograph)***				
Post-treatment	MD	2 (65:74)	−5.06 (−9.55, −0.57), *p* = 0.03	0%
6 months	MD	2 (56:59)	−4.39 (−9.11, 0.33), *p* = 0.07	0%
***Sleep – wake after sleep onset (polysomnograph)***				
Post-treatment	MD	2 (65:74)	−33.91 (−51.82, −16.00), *p* = 0.0002	0%
6 months	MD	2 (56:59)	−22.45 (−41.93, − 2.97), *p* = 0.02	0%
***Sleep - Total sleep time (polysomnograph)***				
Post-treatment	MD	2 (65:74)	16.41 (−23.60, 56.42), *p* = 0.42	29%
6 months	MD	2 (56:59)	18.63 (−12.36, 49.62), *p* = 0.24	0%
***Sleep - Epworth Sleepiness Scale***				
Post-treatment	MD	2 (22:19)	−0.58 (−3.30, 2.14), *p* = 0.68	0%
***Pain***				
Post-treatment	SMD	9 (192:178)	−0.24 (− 0.45, − 0.03), *p* = 0.02	0%
3 months	SMD	3 (83:83)	−0.31 (− 0.69, 0.07), *p* = 0.11	31%
6 months	SMD	3 (66:70)	0.07 (−0.27, 0.41), *p* = 0.68	0%
***Anxiety***				
Post-treatment	SMD	3 (53:53)	−0.54 (−1.01, − 0.06), *p* = 0.03	28%
***Depression***				
Post-treatment	SMD	6 (123:113)	−0.57 (−1.05, − 0.08), *p* = 0.008	65%
3 months	SMD	2 (48:43)	−0.37 (− 0.79, 0.05), *p* = 0.08	0%
**Cognitive behavioural therapy for insomnia versus sleep hygiene**				
***Sleep quality from questionnaire***				
Post-treatment	SMD	3 (65:64)	−0.25 (− 0.82, 0.33), *p* = 0.40	61%
6 months	SMD	2 (33:27)	−0.12 (− 0.84, 0.59), *p* = 0.73	34%
***Pain***				
Post-treatment	SMD	3 (65:64)	−0.51 (−1.17, 0.15), *p* = 0.13	70%
6 months	SMD	2 (33:27)	0.54 (−1.30, 2.38), *p* = 0.57	85%
***Health related quality of life***				
Post-treatment	SMD	2 (50:47)	−0.79 (−1.20, − 0.37), *p* = 0.0002	0%
***Anxiety***				
Post-treatment	SMD	2 (50;47)	−0.32 (− 0.72, 0.08), *p* = 0.12	0%
***Depression***				
Post-treatment	SMD	2 (50;47)	−0.61 (−1.05, − 0.18), p = 0.006	11%
**Cognitive behavioural therapy for insomnia and pain versus no treatment, wait list or attentional control**				
***Sleep quality from questionnaire***				
Post-treatment	SMD	4 (180:192)	−0.79 (−1.58, 0.00), *p* = 0.05	88%
3 months	SMD	2 (56:56)	−0.41 (−1.96, 1.15), *p* = 0.61	94%
6 months	SMD	2 (142:150)	−0.76 (−1.85, 0.33), *p* = 0.17	92%
***Sleep - Total sleep time (diary)***				
Post-treatment	MD	2 (40:34)	−61.58 (−105.25, − 17.91), *p* = 0.006	0%
***Pain***				
Post-treatment	SMD	4 (180:182)	−0.13 (−0.36, 0.10), *p* = 0.28	10%
3 months	SMD	2 (56:56)	−0.48 (− 0.86, − 0.10), *p* = 0.01	0%
6 months	SMD	3 (161:395)	−0.25 (− 0.62, 0.13), *p* = 0.20	58%
***Health related quality of life***				
Post-treatment	MD	2 (58:65)	−1.77 (−5.33, 1.78), *p* = 0.33	98%
3 months	MD	2 (54:49)	−1.84 (−5.90, 2.22), *p* = 037	98%
***Depression***				
Post-treatment	SMD	2 (33:42)	0.14 (−0.54, 0.83), *p* = 0.68	33%
**Cognitive behavioural therapy for insomnia and pain versus cognitive behavioural therapy for pain**				
***Sleep quality from questionnaire***				
Post-treatment	SMD	3 (146:150)	−0.47 (−1.19, 0.25), *p* = 0.20	75%
***Pain***				
Post-treatment only	SMD	3 (146:150)	−0.03 (− 0.26, 0.20), *p* = 0.78	0%
***Heath related quality of life***				
Post-treatment	SMD	2 (139;145)	0.03 (−0.20, 0.26), *p* = 0.81	0%
***Depression***				
Post-treatment	SMD	2 (33:33)	−0.34 (−1.05, 0.37), *p* = 0.35	28%
**Mindfulness versus control**				
***Sleep quality from questionnaire***				
Post-treatment	SMD	2 (105:92)	−0.41 (−0.72, − 0.11), *p* = 0.008	14%
***Pain***				
Post-treatment	SMD	2 (105:92)	−0.35 (− 0.63, − 0.06), *p* = 0.02	0%
***Health related quality of life***				
Post-treatment	SMD	2 (105:92)	−0.28 (− 0.81, 0.26), *p* = 0.31	72%
**Sleep hygiene versus standard care/waitlist control**				
***Sleep quality from questionnaire***				
Post-treatment	SMD	2 (35:26)	−0.68 (−2.37, 1.02), *p* = 0.43	89%
***Pain***				
Post-treatment only	SMD	2 (35:26)	−0.22 (− 0.93, 0.49), *p* = 0.54	45%
**Group-based exercise versus control**				
***Sleep (various overall measures)***				
Post-treatment	SMD	3 (266:240)	−0.10 (− 0.31, 0.12), *p* = 0.39	27%
***Pain***				
Post-treatment	SMD	3 (142:125)	−0.52 (− 0.76, − 0.27), *p* < 0.00001	0%
***Anxiety***				
Post-treatment	SMD	2 (91:81)	−0.51 (−1.43, 0.40), *p* = 0.27	86%
***Depression***				
Post-treatment	SMD	2 (91:81)	−0.41 (−1.26, 0.44), *p* = 0.35	85%
**Tai Ji Quan versus education**				
***Sleep quality from questionnaire***				
Post-treatment	MD	2 (45:45)	−0.78 (−2.31, 0.76), *p* = 0.32	0%

*MD* Mean difference, *SMD* Standardised mean difference

### Psychological interventions

#### Cognitive behavioural therapy for insomnia (CBT-I) versus control

In 10 studies including 482 participants, CBT-I was compared with no treatment, attentional control or wait list control [39–48]. Causes of chronic pain were fibromyalgia (2 studies) [41, 43], osteoarthritis (2 studies) [45, 48], spinal pain (1 study) [42], multiple sclerosis (1 study) [39], migraine (1 study) [46], and diverse chronic pain (3 studies) [40, 44, 47]. Three studies were judged to be at low risk of bias [42, 45, 46], but no studies were considered to be at high risk.

**Fig. 2:**
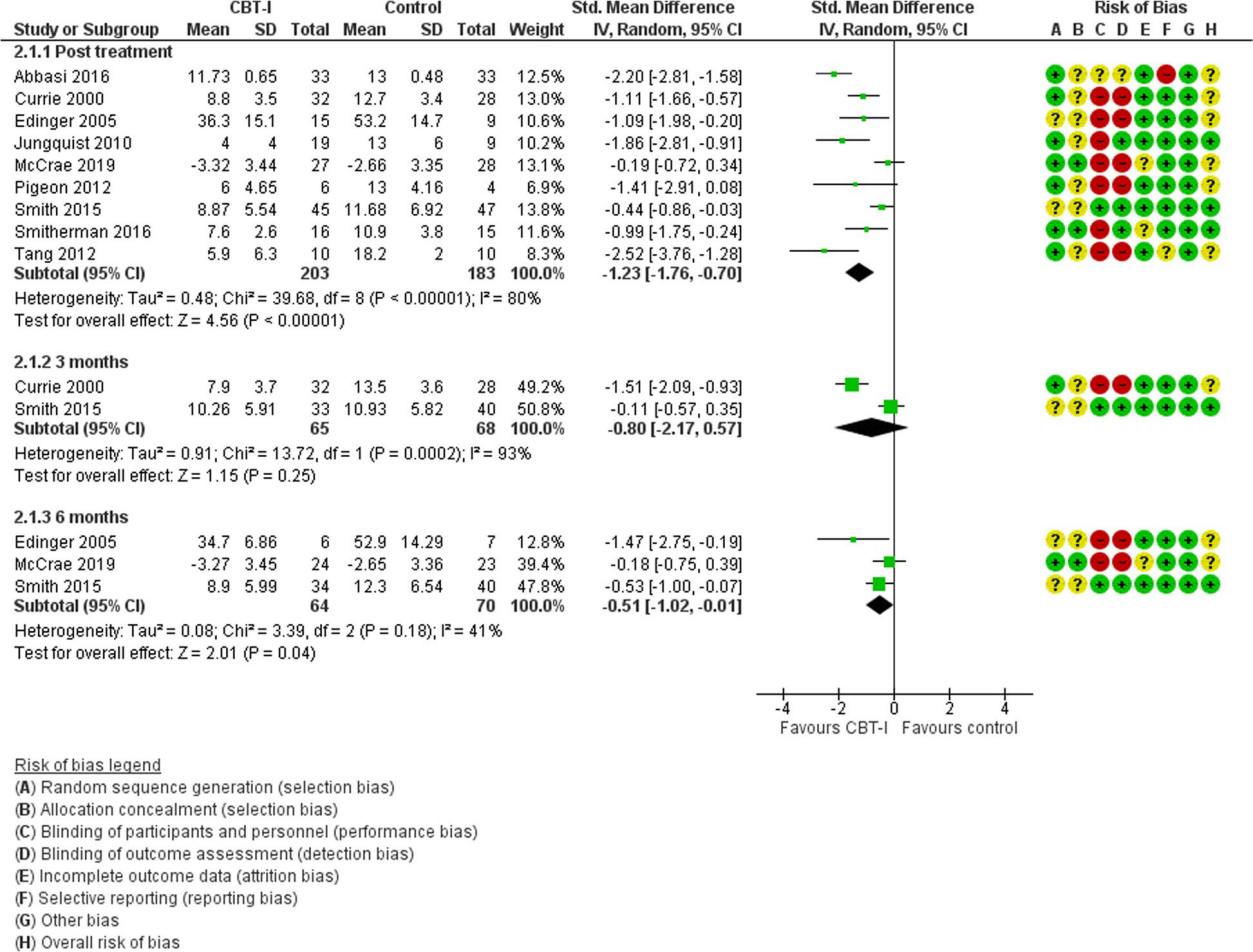
CBT-I versus control. Sleep quality

At the end of intervention, overall questionnaire assessed sleep quality in 9 studies with 385 participants was improved in people receiving CBT-I compared with untreated controls, SMD -1.23 (95%CI -1.76, − 0.70), *p* < 0.00001 (Fig. 2). Heterogeneity was high, (I^2^ 80%). The improvement was sustained but reduced at 6 months in 3 studies with data. In 3 studies at low risk of bias, the benefit for CBT-I over control was slightly reduced, SMD -1.01 (95%CI -1.79, − 0.22), *p* = 0.01 and heterogeneity remained high (I^2^ 74%). Exploration of effectiveness in relation to a specific condition was only possible for 2 studies at unclear risk of bias including 79 people with fibromyalgia suggesting no benefit for CBT-I, SMD -0.57 (95%CI -1.44, 0.30), *p* = 0.2, but heterogeneity was high (I^2^ 65%).

Waking after sleep onset, as measured by actigraphy, improved in people receiving CBT-I post-treatment compared with controls and this was also apparent up to at least 6 months in those studies using sleep diaries and polysomnography. Sleep onset latency was improved up to 3 months after CBT-I when assessed by questionnaire, but not with actigraphy or polysomnography. Sleep efficiency was improved up to 6 months after CBT-I compared with control when measured in sleep diaries or by polysomnography, but not by actigraphy. Total sleep time measured by diary, actigraphy or polysomnography was not improved in those receiving CBT-I compared with controls. There were no improvements in diary recorded sleep awakenings or the Epworth Sleepiness Scale in people receiving CBT-I compared with control. Adverse events were assessed in 5 studies [42, 43, 45–47]. No adverse events were reported in 4 studies. In 1 study 3 adverse event cases were deemed to be study related [45], this included rash from wearing actigraph and tenderness at site of testing.

**Fig. 3:**
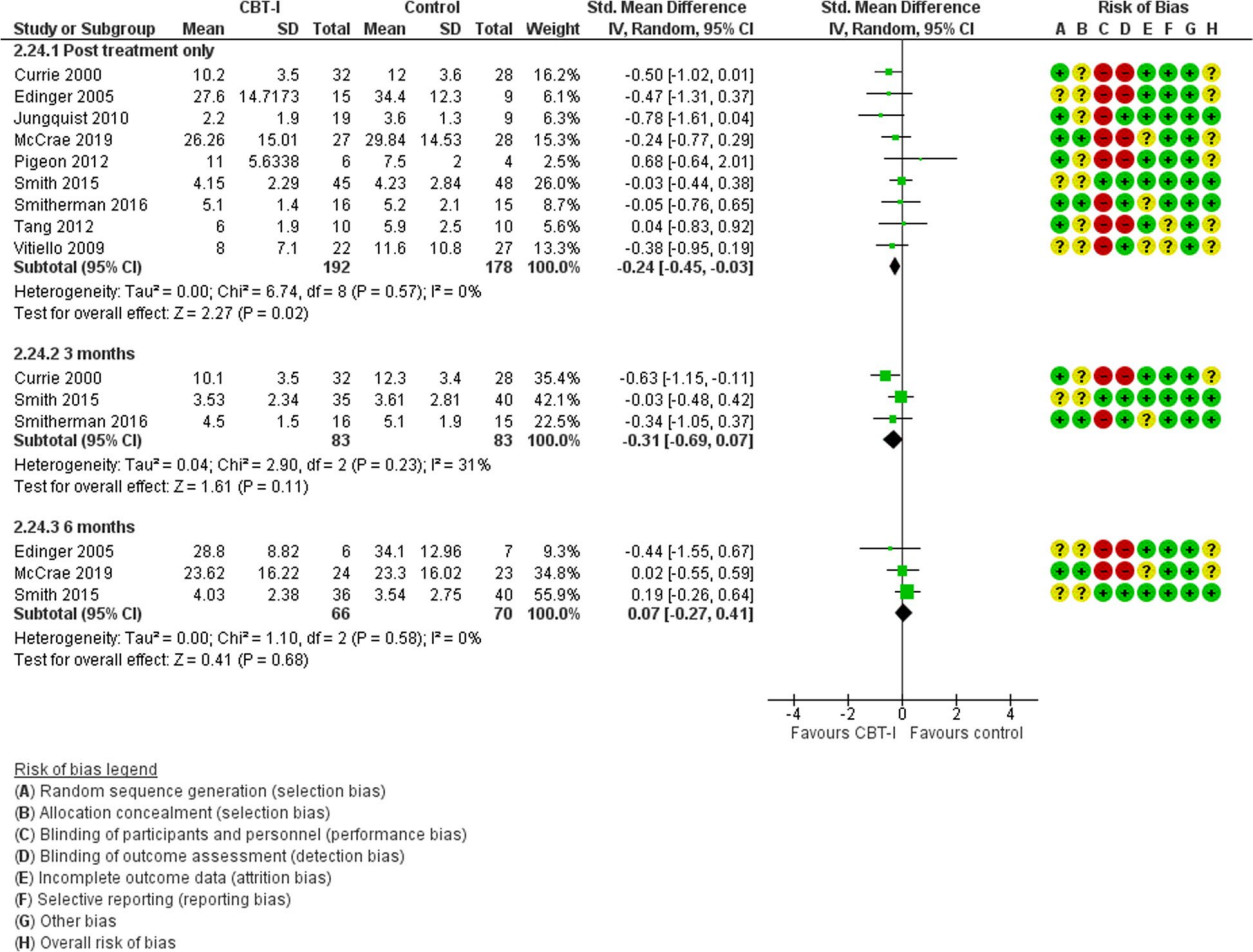
CBT-I versus control. Pain

In 9 studies with 370 people randomised, pain measured by questionnaire was reduced post-treatment in people receiving CBT-I compared with controls, SMD -0.24 (95%CI -0.45, − 0.03; *p* = 0.02), but this was not apparent in 3 studies at low risk of bias, SMD -0.19 (95%CI -0.59, 0.220), *p* = 0.36, or sustained at 3 months or longer (Fig. 3). There was no evidence of heterogeneity. In 2 studies at unclear risk of bias including 79 people specifically with fibromyalgia, there was no benefit for CBT-I, SMD -0.31 (95%CI -0.75, 0.140), *p* = 0.18. There was no suggestion of heterogeneity.

No studies reported health-related quality of life outcomes. For psychological health, information from studies was mainly limited to post-treatment with benefit suggested for anxiety SMD -0.54 (95% CI -1.01, − 0.06), *p* = 0.03 with slight heterogeneity (I^2^ 28%), and depression SMD -0.57 (95%CI -1.05, − 0.08), *p* = 0.08 with high heterogeneity (I^2^ 65%). IN 1 study exclusively including people with fibromyalgia, anxiety and depression were reduced in the Group receiving CBT-I compared with controls.

In 2 studies, a further comparison was made between CBT-I and CBT solely for pain (CBT-P). One was a small pilot study with 11 people with chronic pain randomised to the 2 interventions [44]. In a larger study with unclear risk of bias due to high losses to follow up, there was no suggestion of benefit for any outcome for CBT-I compared with CBT-P in people with fibromyalgia [43].

#### Brief education with CBT component versus no treatment

In 1 study with 132 people with chronic non-cancer pain randomised, a brief educational intervention incorporating sleep hygiene and cognitive behavioural strategies was compared with wait list controls [49]. The study was at high risk of bias due to large losses to follow up. Only sleep outcomes were reported and there was no difference between groups in sleep quality or sleep diary measures excepting diary recorded sleep latency which favoured the intervention.

#### Cognitive behavioural therapy for insomnia (CBT-I) versus sleep hygiene

CBT-I was compared with a sleep hygiene intervention in 4 randomised trials [41, 50–52]. Studies included 270 participants, all with chronic pain from fibromyalgia. Risk of bias was low in 3 studies [50–52] and unclear in 1 due to limited reporting of methods [41].

Data on overall sleep quality was available for 3 studies [41, 50, 51]. There was no difference between randomised groups post-treatment, SMD -0.25 (95%CI -0.82, 0.33), *p* = 0.40 but heterogeneity was high (I^2^ 61%). Excluding the study at unclear risk of bias removed heterogeneity and there was an improvement in overall sleep quality after CBT-I compared with sleep hygiene, SMD -0.53 (95%CI -0.94, − 0.12), *p* = 0.01 [50, 51]. Evidence relating to longer term outcomes was limited but with no clear suggestion of benefit for CBT-I over sleep hygiene.

In 3 studies, results for pain outcome were similar in direction to sleep quality [41, 50, 51] but a difference favouring CBT-I post-treatment was only apparent in the 2 studies at low risk of bias, SMD -0.85 (95%CI -1.26, − 0.43), *p* < 0.0001 [50, 51] with no evidence of heterogeneity. Health-related quality of life was improved in people receiving CBT-I compared with sleep hygiene in 2 studies with 97 people randomised, both at low risk of bias and with no heterogeneity, SMD -0.79 (95%CI -1.20, − 0.37), *p* = 0.0002. Improvements in pain and health-related quality of life were not evident at longer follow up. Evidence relating to psychological health was limited to 2 studies at low risk of bias with 97 patients randomised. There was no benefit for CBT-I compared with sleep hygiene for anxiety, SMD -0.32 (95%CI -0.72, 0.08), *p* = 0.12 with no heterogeneity, but depression was reduced, SMD -0.61 (95%CI-1.05, − 0.18), *p* = 0.006 with slight heterogeneity (I^2^ 11%).

#### Cognitive behavioural therapy for insomnia and pain (CBT-IP) versus control

**Fig. 4:**
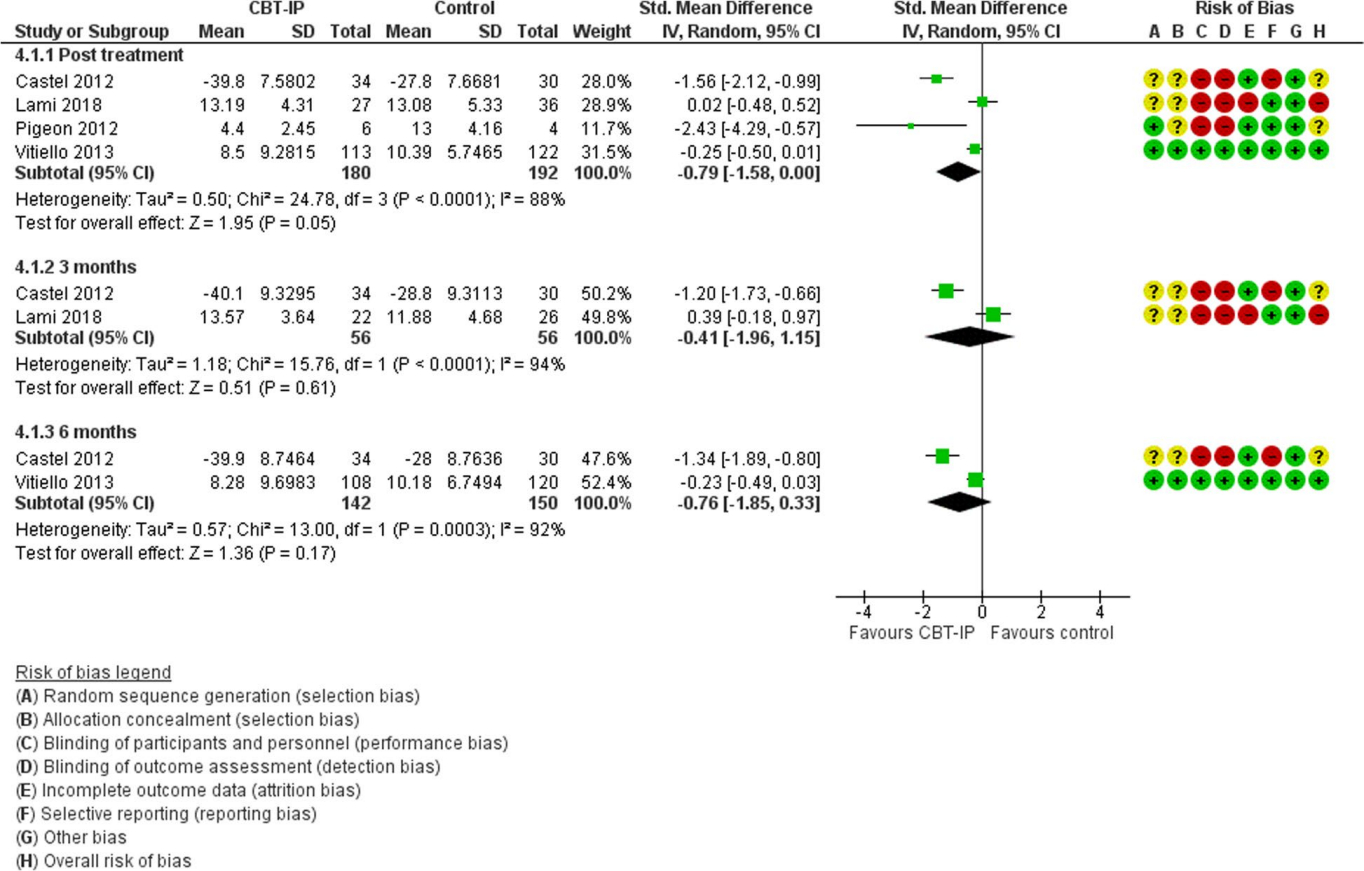
CBT-IP versus control. Sleep quality

In 4 studies with 432 participants randomised, cognitive behavioural therapy focusing on insomnia and pain (CBT-IP) was compared with no treatment, wait list or attentional control [44, 53–55]. In 2 studies, the cause of pain was fibromyalgia [53, 54], and in 1 each, osteoarthritis [55], or diverse causes [44]. Risk of bias was high in 1 study due to large losses to follow up at the end of treatment [54], and unclear in 1 due to lack of methodological detail [53]. A third was a small pilot study [44]. For sleep quality, data for meta-analysis was available from all studies post-treatment (Fig. 4). Compared with controls, people receiving CBT-IP had marginally improved sleep quality and improved diary recorded total sleep time, SMD -0.79 (95%CI -1.58, 0.00), *p* = 0.05, and MD − 61.58 min (95%CI -105.25, − 17.91), *p* = 0.006, respectively. In the 1 study at low risk of bias, the difference in sleep quality was smaller and in 2 studies with data, the benefit relating to sleep quality was not sustained at 3 and 6 months. In 2 studies with 299 people exclusively with fibromyalgia, there was no evidence for a difference in sleep quality, SMD -0.88 (95%CI -2.16, 0.41), *p* = 0.18 but heterogeneity was high (I^2^ 94%).

**Fig. 5:**
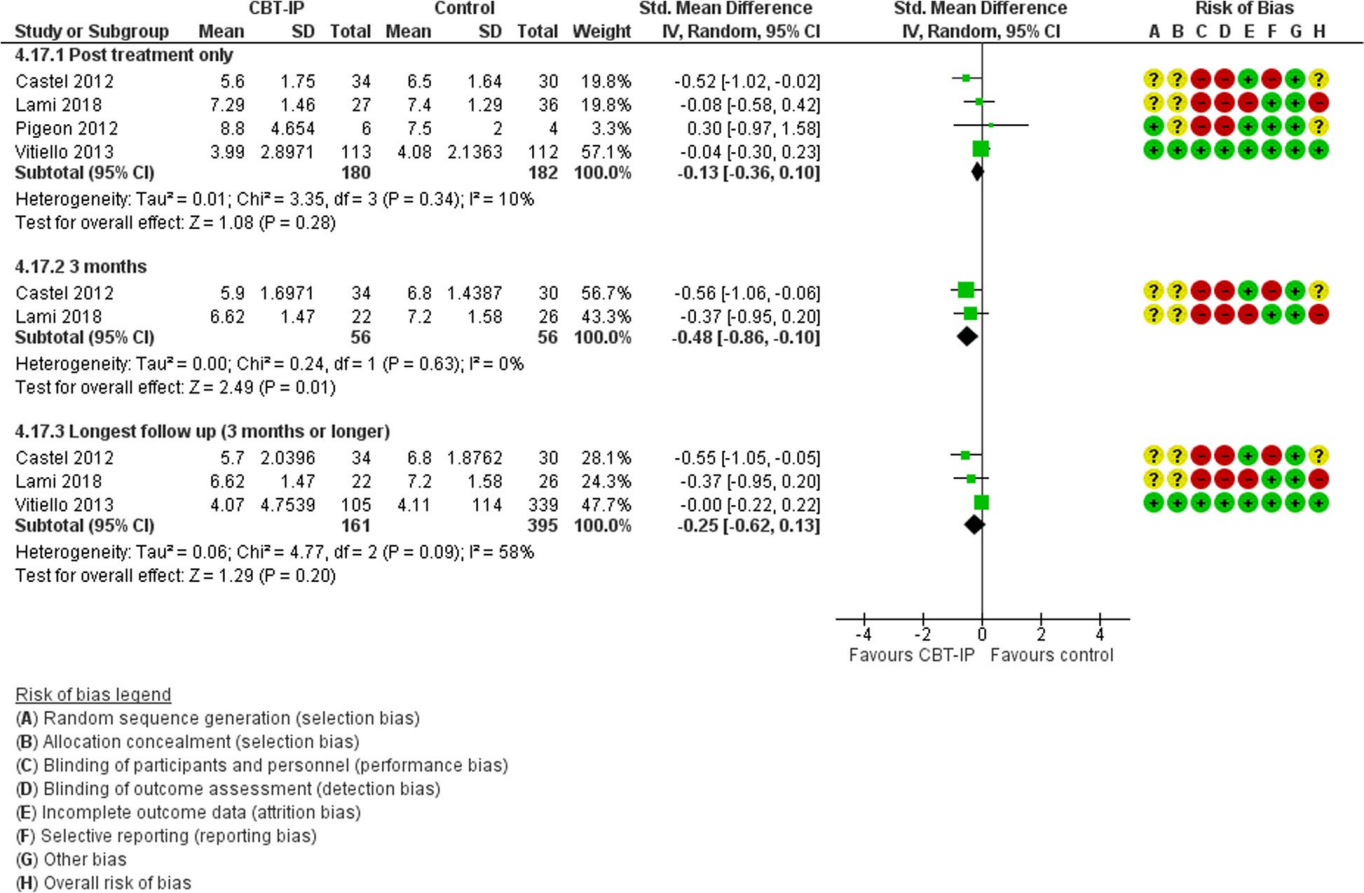
CBT-IP versus control. Pain

All 4 studies reported a pain outcome post-treatment (Fig. 5) [44, 53–55]. There was no benefit for CBT-IP compared with control except in 2 studies at 3 months [53, 54]. No benefit was seen in the 2 studies of people with fibromyalgia or the study at low risk of bias [55]. Information was limited relating to health-related quality of life and psychological health but in 2 studies there was no difference post-treatment in quality of life or depression between groups.

In 4 studies with 415 people randomised, CBT-IP was compared with a control condition that included cognitive behavioural therapy for pain (CBT-P) with no specific focus on sleep [43, 44, 54, 55]. In studies with data suitable for meta-analysis there was no difference in sleep quality, pain or health-related quality of life post-treatment, or anxiety or depression post-treatment or at 3 months, and this was not changed if restricted to studies at low or unclear risk of bias or in a study exclusively including people with fibromyalgia. Heterogeneity was high (I^2^ 75%).

In 1 study at unclear risk of bias due to limited reporting of methods with 95 people with fibromyalgia randomised, hypnosis additional to CBT-IP was evaluated [53]. Compared with CBT-IP alone, there were no differences in sleep quality, diary assessed total sleep time, or pain post-treatment and at 3 and 6 months. Health-related quality of life was improved after CBT-IP with hypnosis compared with CBT-IP but only post-treatment and at 3 months. At 6 months, CBT-IP without hypnosis showed a favourable outcome. General psychological health was improved in people receiving CBT-IP and hypnosis compared with CBT-IP post-treatment and at 3 and 6 months.

#### Acceptance and commitment therapy versus attentional control or exercise

In 1 study, 299 people with chronic pain were randomised to a 7-week course of acceptance and commitment therapy based stress management, or to a control discussion group of similar intensity and duration, or to group-based exercise [56]. The study was at high risk of bias due to high losses to follow up. There were no differences between randomised groups in insomnia severity, pain, anxiety or depression at the end of treatment or at 6 month follow up.

#### Mindfulness versus control

In 2 studies including 239 people with fibromyalgia pain, a mindfulness-based intervention was compared with waiting list controls [57], or CBT with no specific focus on sleep [58]. Risk of bias was unclear in the former mainly because of wait list controls [57], while in the latter, risk of bias was high mainly due to large losses to follow up [58]. In meta-analysis, there was benefit for improved sleep quality post-treatment after mindfulness intervention compared with wait list controls or CBT, SMD -0.41 (95%CI -0.72, − 0.11), *p* = 0.008, and this was consistent at 3 [57], and 6 months [58]. For pain, and health-related quality of life, there was no consistent evidence for benefit of mindfulness intervention over CBT or wait list control.

#### Relaxation versus control

In 1 study with 53 people with fibromyalgia pain, group and individualised applied relaxation was compared with a sleep hygiene-based educational intervention, and a wait list control [59]. Risk of bias was low for the comparison of interventions, but unclear in relation to the wait list control. There were no differences post-treatment between sleep quality, pain, or health-related quality of life in relaxation and wait list control groups. This was also the case for relaxation compared with education, with the exception of improved pain in the relaxation group at 6 month follow up. Risk of bias was high at this follow up time due to high and uneven losses to follow up.

### Sleep hygiene versus control

Further to the studies comparing sleep hygiene intervention with CBT-I [41, 50–52], in 2 studies with 54 people with fibromyalgia pain, sleep hygiene was compared with untreated controls [41, 59]. Risk of bias in 1 study with a wait list control was unclear [59], and high in the other due to uneven randomisation and large loss to follow up [41]. In meta-analysis, there was no benefit post-treatment for sleep hygiene compared with controls for sleep quality or pain (Table 2). A difference in general psychological health favouring sleep hygiene over control was limited to a single study at high risk of bias.

### Physical exercise

#### Group-based exercise versus control

In 5 studies with 697 people randomised, exercise programmes were compared with usual care or an attentional control [56, 60–63]. Pain conditions were, arthritis, rheumatoid arthritis, fibromyalgia, low back pain and general chronic pain. In 4, the programme was delivered at a clinic or in a group [56, 60, 62, 63], and in 1 at home [61]. Studies were at low [61], unclear [60, 62, 63], or high risk [56] of bias. One was a feasibility study [62]. For the 3 studies with data [56, 61, 63], questionnaire assessed sleep quality was not improved in the exercise groups compared with controls, SMD -0.10 (95%CI -0.31, 0.12), *p* = 0.39 and this was consistent in the study at low risk of bias. There was no difference in 1 study with sleep measures at 6 months [56]. One study including 321 people with arthritis comparing a low to moderate intensity physical activity programme with wait list controls and at unclear risk of bias, presented sleep data dichotomised into groups of people with no problems and those with moderate to severe problems [63]. In intention to treat analyses, the authors reported post-treatment benefit for the intervention compared with controls for the outcome waking up tired, *p* < 0.001, but not trouble falling asleep or staying asleep, waking up at night, or trouble staying asleep. Benefit was not maintained at 3 or 6 months. In one study including 53 people with fibromyalgia, the authors reported that sleep quality was improved in the group receiving group-based exercise compared with controls, *p* = 0.051 [56, 60].

Pain in 3 studies was reduced in the exercise group compared with controls, SMD -0.52 (95%CI -0.76, − 0.27), *p* < 0.00001 [56, 60, 61], and this was consistent in the study at low risk of bias [61] and as reported by the authors in the study specifically in people with fibromyalgia, *p* = 0.039 [56, 60]. In 2 studies reporting anxiety and depression, there was no benefit for exercise compared with controls [56, 60]. The study with dichotomised outcomes showed no benefit for physical activity intervention in relation to health-related quality of life or mental health [63]. Adverse events were assessed in 1 study [62] with 2 events reported, increase in low back pain (*n* = 1) and increase in knee pain (*n* = 1).

#### Home-based walking programme versus exercise or control

In a feasibility study with 60 people with chronic low back pain randomised, a walking intervention was compared with supervised exercise and controls [62]. Risk of bias was unclear due to lack of blinding. While no differences were apparent in sleep quality, pain, general psychological health, anxiety or depression, the authors concluded that their screening methods and new intervention could be evaluated in a fully powered trial.

#### Comparison of exercise interventions

In 2 studies, different exercise modalities were compared [64, 65].

In a pilot study, 40 people with multiple sclerosis pain were randomised to a clinic-based moderate-intensity aerobic exercise programme or low-intensity home-exercise programme [64]. Moderate-intensity aerobic exercise improved sleep quality and Actigraph sleep measures post-treatment compared with the low-intensity exercise. However, the study was at high risk of bias due to high and uneven loss to follow up.

A course of aquatic biodance was compared with a course of stretching exercises in 1 study with 76 people with fibromyalgia pain [65]. The randomised trial was at unclear risk of bias due to high losses to follow up at end of treatment, but the analysis reported was intention to treat. Immediately after treatment, sleep quality, pain, health-related quality of life, anxiety and depression were all improved in the group who participated in aquatic biodance compared with those who did stretching exercises.

#### Tai Ji Quan versus education

In 2 studies with 90 people randomised, Tai Ji Quan was compared with an education intervention [66, 67]. Chronic pain conditions included were knee osteoarthritis [66] and fibromyalgia [67]. One study was at low risk of bias [66] and 1 was at unclear risk due to limited reporting [67]. In meta-analysis there was little difference in sleep quality post-treatment, SMD -0.78 (95%CI -2.31, 0.76), *p* = 0.32 and this was consistent in the study at low risk of bias. No heterogeneity was evident. Also in the study at low risk of bias, sleep efficiency measured by questionnaire and diary was improved in people receiving Tai Ji Quan [66], but there were no differences in other sleep, pain or psychological measures.

### Physical therapy

Physical therapy modalities were evaluated in 6 studies.

#### Hydrotherapy

Two studies described treatment comparisons including hydrotherapy [68, 69]. In 1 study including 81 people with fibromyalgia pain, a course of hydrotherapy with stretching was compared against hydrotherapy with Tai Chi [68]. Risk of bias was high due to large losses to follow up. Results were presented as graphs and interpreted by the authors as showing no differences between randomised groups.

A course of hydrotherapy was compared with conventional physiotherapy in 1 study at low risk of bias with 50 people with fibromyalgia randomised [69]. Post-treatment, total sleep time was marginally higher and total nap time lower in people who received hydrotherapy compared with conventional physiotherapy. Pain and general psychological health did not differ between groups post-treatment. Adverse events were assessed in 1 study [68], 3 patients dropped out of the intervention group due to pain exacerbation (*n* = 2) and chlorine hypersensitivity (*n* = 1).

#### Massage or manual therapy

A course of massage therapy was compared with relaxation therapy in 1 study with 30 people with low back pain [70]. Reporting was limited and risk of bias unclear. A large difference in sleep disturbance between groups was apparent at baseline. Post-treatment, there were no differences between groups. A possible favourable sleep disturbance outcome for massage therapy at follow up may have been masked by the difference at baseline.

In 1 study with 89 people with fibromyalgia pain randomised, a course of manual therapy was compared with no treatment [71]. The study was at unclear risk of bias due to lack of blinding of the intervention. Results reported separately for men and women suggested improved sleep quality, pain, health-related quality of life and depression in people who received manual therapy compared with controls.

#### Physical therapy programme

In 1 study including 60 people with fibromyalgia, a physical therapy programme with hot pack, ultrasound, transcutaneous electrical nerve stimulation and low power laser was compared with an untreated control group [72]. Risk of bias was high mainly through lack of blinding. The authors reported improvements in sleep quality, pain and health-related quality of life in people receiving the physical therapy programme compared with controls.

#### Pompage

A course of pompage was compared with controls in 1 study with 23 people with fibromyalgia pain randomised [81]. All participants received stretching and aerobic exercises. Risk of bias was high to large losses to follow up. There was no improvement in sleep quality or pain post-treatment in people receiving pompage compared with controls.

### Other interventions

#### Acupressure

Auricular point acupressure was compared with sham auricular point acupressure in 1 study including 61 people with chronic low back pain [74]. Risk of bias was high due to large and uneven losses to follow up. People receiving active intervention reported improved sleep quality post-treatment compared with the sham group. Differences in other sleep measures were marginal. In another study, self administered relaxing or stimulating acupressure was compared with usual care in 67 people with low back pain [75]. Risk of bias was unclear due to limited reporting of methods. With results shown as graphs, the authors reported no improvement in sleep quality between acupressure groups, and compared with controls. Pain was reduced in acupressure groups but not in controls. Adverse events were assessed 1 study [75] with four mild events related to acupressure treatments involving too much pressure to acupoints.

#### Bright light treatment

In 1 study, visor delivered bright light treatment was compared with sham therapy in 19 people with fibromyalgia pain randomised in a crossover trial [76]. The study was at high risk of bias due to large losses to follow up. There were no differences between groups in sleep quality, hours slept per night, awakenings per night, pain, anxiety or depression.

#### Foot reflexology versus control

In 1 study, 68 people with pain from rheumatoid arthritis were randomised to a course of foot reflexology or control [77]. Risk of bias was unclear due to lack of blinding. Actigraphy was only completed for 25% of participants. Sleep quality and pain were improved in people receiving reflexology compared with controls.

#### Transcranial stimulation versus sham

One study compared transcranial stimulation with sham in 16 patients with musculoskeletal pain [78]. This was a small feasibility study with unclear risk of bias due to limited reporting. People receiving transcranial stimulation had reduced pain after the intervention compared with controls, but there was no difference in sleep quality. The authors described aspects of study conduct to advise future evaluations of transcranial stimulation.

#### Mattress interventions versus control

In 1 study at low risk of bias with 30 people with fibromyalgia pain randomised, a period of sleep on a magnetic mattress pad was compared with sham [79]. Sleep, pain and ADL focused quality of life were improved in the intervention group compared with sham control. Adverse events were assessed with none reported. A period of sleep on a supportive mattress overlay was compared with untreated controls in 1 study with 38 people with low back pain randomised [80]. The study was at high risk of bias due to concerns about the randomisation procedure and blinding. Post-treatment, sleep quality was similar between groups. Presented as graphs, the authors reported improved pain in the mattress overlay group compared with controls but no difference in sleep quality.

## Discussion

We identified 42 randomised trials evaluating sleep interventions for people with chronic pain. CBT interventions provided the largest evidence base with CBT-I interventions demonstrating benefit post-treatment compared with controls for improved sleep quality, however evidence for a longer-term sustained benefit was lacking. Evidence in people with specific conditions was limited due to primary study limitations and statistical heterogeneity. Numerous interventions were evaluated in small numbers of studies, but evidence was insufficient to draw conclusions on effectiveness.

Findings from meta-analysis found that CBT-I (9 studies) and CBT-IP (4 studies) demonstrated a medium to large (− 0.79) effect compared with control for sleep quality at post-treatment. Differences between groups at 6 months was slightly reduced with a medium effect size for CBT-I and was not sustained for CBT-IP. CBT-I showed a small improvement in pain outcome post-treatment but this was not sustained. However, there was high heterogeneity in studies which should be considered when interpreting results. In 4 studies comparing CBT-IP with CBT-P only, no differences in pain outcome were found. Due to the active control it is therefore only possible to infer that adding insomnia specific content to CBT-P does not have an additional impact on pain outcomes. Our findings regarding the impact of CBT-I interventions on sleep quality reflect the existing literature. A recent systematic review of CBT-I therapies in patients with chronic non-malignant pain showed significant treatment effects immediately post-treatment for global measures of sleep [73]. Condition specific reviews show similar results with CBT therapies improving sleep outcomes in the short-term for patients with lower back pain, fibromyalgia, and osteoarthritis [82–84].

Our results demonstrate that improving sleep for people with chronic pain is possible, and that CBT approaches have the strongest evidence base. Poor sleep has a negative impact on optimism, sociability, and psychosocial functioning [85]. Poor sleep also has clear links with depression and pain catastrophising, both of which can affect pain management and coping. Pain catastrophising is linked with maladaptive coping techniques and depression is linked to lack of engagement in treatment [86, 87]. CBT approaches are already widely used in pain management with a focus on coping strategies and behavioural rehearsal [88]. This systematic review demonstrates that additional focus on sleep improvement could be of benefit. As CBT is an established treatment approach, future work should focus on how best to implement CBT sleep interventions for people with chronic pain and foster more equitable access to support, particularly for underserved populations. Results from the National Pain audit highlighted that service provision for the management of chronic pain in the UK is inadequate [89]. Only 40% of pain clinics in the UK are multidisciplinary which presents a challenge for implementation of psychological interventions such as CBT.

Evaluation of the longer-term effectiveness of sleep interventions for people with chronic pain is lacking. Although evidence suggests that CBT interventions improve sleep immediately post treatment, effects reduce over time. In addition, most studies had follow-up data collected at 3 months or less post-intervention, with 9 studies reporting 6-month outcomes, and 2 studies reporting 12-month outcomes. Understanding longer-term effectiveness of these interventions is crucial for people with chronic pain. Due to the nature of the condition, individuals with chronic pain may experience disturbed sleep for many months and years, therefore effective interventions need to have sustained effects.

Assessment of outcomes within the trials included in this review varied considerably, and this was particularly notable for the secondary outcomes. This heterogeneity limits comparison between studies, particularly for health-related quality of life and psychological wellbeing, because outcome measures assess different aspects of these constructs, for example general mood assessment versus specific anxiety or depression measures. The issue of heterogeneity across randomised trials is well established and initiatives such as COMET have been addressing this through the development of core outcome sets [90, 91]. Core outcome sets provide a minimum set out of outcomes to be used in all trials of a certain focus, ensuring comparability across multiple studies. The challenge of a review of this scope is that the interventions included are varied and include both generic and condition specific measures. As the evidence base builds, focused reviews on areas of promise, such as CBT and third wave therapies, could offer benefit. The feasibility of developing a sleep core outcome set could also be explored as this would be provide opportunity for greater comparison across interventions.

Nine of the 42 studies in this review provided data on adverse events with no serious adverse events reported [42, 43, 45–47, 62, 68, 75, 79]. Assessment of adverse events is vital for patient safety, however unlike in trials of pharmacological treatments where monitoring and reporting of harm outcomes is mandatory, behavioural and psychological interventions are not held to the same account [92]. In 2004 the CONSORT group provided ten recommendations for reporting harm outcomes in trials [93]. All except 5 of the studies included in our review were published after the recommendations. This demonstrates a need for evaluations of psychological and behavioural interventions to improve reporting of harm outcomes.

### Strengths and limitations

This review has strengths and limitations that should be considered when interpreting the findings. The method used in this review was robust and systematic, following Cochrane guidance [36]. The review provides a comprehensive overview of the existing literature on sleep interventions for patients with chronic pain, but as included studies addressed a wide variety of interventions with small numbers of studies for each intervention, this limited opportunity for meta-analysis. The population of people with chronic pain that we considered is heterogenous with a range of underlying medical conditions. However, chronicity reflects pain that persists for 3 months or longer, and chronic pain is usually the affected person’s main clinical problem. Secondary outcome measure tools used were highly heterogenous, which limited comparison between studies.

## Conclusion

CBT approaches have the potential to be an effective treatment to improve sleep for people with chronic pain, but further high-quality primary research is required to explore refinements that will ensure parallel improvements to pain, quality of life, psychological health and maintain all benefits in the long term. Individuals who experience depression and pain catastrophising may particularly benefit from sleep interventions. As CBT is an established treatment approach, future work should focus on how best to deliver these interventions, for instance by exploring any difference between online or face-to-face delivery or differences between delivery professionals. Importantly, future research could focus on how best to facilitate equal access and outcomes in underserved populations.

Primary research is also needed to evaluate the effectiveness of interventions including mindfulness, aquatic exercise and hydrotherapy, Tai Ji Quan, manual therapy, physical therapy programmes, acupressure, foot reflexology and magnetic mattress pads. Individuals who experience chronic pain could benefit from interventions that address sleep, and research is needed to assess any impact of sleep interventions on pain.

## Supplementary Information

**Additional file 1.**

**Additional file 2.**

**Additional file 3.**

**Additional file 4.**

**Additional file 5.**

## Data Availability

No additional data are available. Extracted data is included within the manuscript and supplementary materials.
