# Supplementary material for: The effectiveness of non-pharmacological sleep interventions for people with chronic pain: a systematic review and meta-analysis
Source: BMC Musculoskelet Disord. 2022 May 11;23:440. doi: 10.1186/s12891-022-05318-5 (PMC9092772; doi:10.1186/s12891-022-05318-5)
Supplement: Supplementary file 2 — Additional file 2. [file 12891_2022_5318_MOESM2_ESM.docx]

**Supplement. Table2: Search strategy as applied in MEDLINE on 8^th^ April 2020**

1 controlled clinical trial.pt.

2 randomized controlled trial.pt.

3 clinical trials as topic/

4 (randomi#ed or randomi#ation or randomi#ing).ti,ab,kf.

5 (RCT or "at random" or (random* adj3 (administ* or allocat* or assign* or class* or cluster or crossover or cross-over or control* or determine* or divide* or division or distribut* or expose* or fashion or number* or place* or pragmatic or quasi or recruit* or split or subsitut* or treat*))).ti,ab,kf.

6 placebo.ab,ti,kf.

7 trial.ti.

8 (control* adj3 group*).ab.

9 (control* and (trial or study or group*) and (waitlist* or wait* list* or ((treatment or care) adj2 usual))).ti,ab,kf.

10 ((single or double or triple or treble) adj2 (blind* or mask* or dummy)).ti,ab,kf.

11 double-blind method/ or random allocation/ or single-blind method/

12 or/1-11

13 (systematic or structured or evidence or trials or studies).ti. and ((review or overview or look or examination or update* or summary).ti. or review.pt.)

14 (0266-4623 or 1469-493X or 1366-5278 or 1530-440X or 2046-4053).is.

15 meta-analysis.pt. or (meta-analys* or meta analys* or metaanalys* or meta synth* or meta-synth* or metasynth*).ti,ab,kf,hw.

16 ((systematic or meta) adj2 (analys* or review)).ti,kf. or ((systematic* or quantitativ* or methodologic*) adj5 (review* or overview*)).ti,ab,kf,sh. or (quantitativ$ adj5 synthesis$).ti,ab,kf,hw.

17 (integrative research review* or research integration).tw. or scoping review?.ti,kf. or (review.ti,kf,pt. and (trials as topic or studies as topic).hw.) or (evidence adj3 review*).ti,ab,kf.

18 review.pt. and ((medline or medlars or embase or pubmed or scisearch or psychinfo or psycinfo or psychlit or psyclit or cinahl or electronic database* or bibliographic database* or computeri#ed database* or online database* or pooling or pooled or mantel haenszel or peto or dersimonian or der simonian or fixed effect or ((hand adj2 search*) or (manual* adj2 search*))).tw,hw. or (retraction of publication or retracted publication).pt.)

19 or/13-18

20 exp "Sleep Initiation and Maintenance Disorders"/

21 SLEEP/

22 sleep*.mp.

23 insomnia.mp.

24 exp "Wakefulness"/

25 wakeful*.mp.

26 sleepless*.mp.

27 exp "Dyssomnias"/

28 dyssomn*.mp.

29 or/20-28

30 PAIN/

31 Chronic Pain/

32 Arthritis/ or Osteoarthritis/ or Arthritis, Rheumatoid/ or Arthritis, Psoriatic/

33 Fibromyalgia/

34 Back Pain/

35 Multiple Sclerosis/

36 headache disorders/ or Migraine disorders/

37 or/30-36

38 19 and 29 and 37

39 limit 38 to humans
