# Supplementary material for: The effectiveness of non-pharmacological sleep interventions for people with chronic pain: a systematic review and meta-analysis
Source: BMC Musculoskelet Disord. 2022 May 11;23:440. doi: 10.1186/s12891-022-05318-5 (PMC9092772; doi:10.1186/s12891-022-05318-5)
Supplement: Supplementary file 4 — Additional file 4. [file 12891_2022_5318_MOESM4_ESM.docx]

**Supplement. Table 4: Risk of bias**

| **Study** | **Random sequence generation** | **Allocation concealment** | **Blinding participants and personnel** | **Blind outcome assessment** | **Incomplete outcome data** | **Selective reporting** | **Other bias** | **Summary risk of bias** |
| --- | --- | --- | --- | --- | --- | --- | --- | --- |
| Abbasi et al. 2016[33] | Low -minimisation | Unclear | Unclear (controls received something but less in quantity of sessions) | Unclear | Low | High - anxiety and depression specified as outcomes in protocol | No | Unclear |
| Al-Sharman et al. 2019[58] | Unclear not much detail provided | Unclear not much detail provided | High - main assessor blinded but not patients | Low (both groups received either an intervention or a sham procedure) | High - loss to follow-up 25%. Higher in controls than intervention 35% vs 15% | Unclear | No | High |
| Arcos-Carmona et al. 2011[54] | Unclear | Unclear | Low | Low (groups received either an intervention or sham procedure) | Low | Low - none apparent | Little detail | Unclear |
| Bakir et al. 2018[71] | Low - random number table | Unclear | Low | Unclear | Low | Low - none apparent | No | Unclear |
| Berry et al. 2015[43] | Low | Low | High | High (wait list control) | High 35% of patients excluded from analysis because they did not return all the measures | Low | No | High |
| Calandre et al. 2009 [62] | Low | Unclear | High | High (no intervention in control group) | High - 30% of patients not included in analysis | High - only graphs given | No | High |
| Cash et al. 2015[51] | Low - random number | Low - research associate blinded | High | High (wait list control) | Low | Low - None apparent | No | Unclear |
| Castel et al. 2012[47] | Unclear | Unclear | High | High (no intervention in control group) | Low | High (complete MOS Sleep Scale but only two dimensions reported) | No | Unclear |
| Castro-Sanchez et al. 2014[65] | Low | Low | High | High (no intervention in control group) | Low | Low | No | Unclear |
| Colbert et al. 1999[73] | Low | Low | Low | Low (groups received intervention or sham procedure) | Low | Low | No | Low |
| Correia Moretti et al. 2016[67] | Low | Low | Unclear (both groups received something) | Low (groups received intervention or sham procedure) | High 35% lost to follow up | Low | No | High |
| Currie et al. 2000[34] | Low - random number table | Unclear | High | High (wait list control) | Low | Low | No | Unclear |
| Durcan et al. 2014[57] | Low - Excel | Unclear | Unclear | Low | Low | Low | Baseline difference in pain and stiffness | Low |
| Eadie et al. 2013[55] | Low - computer generated | Low - opaque envelopes | High | High (no intervention in control group) | Low. High risk at 6 months due to high loss to follow up | None apparent | No | Unclear  High risk at 6 months  Feasibility study |
| Edinger et al. 2005[35] | Unclear | Unclear | High | High (no intervention in control group) | Low (high drop out at 6 months) | Low | No | Unclear  High risk at 6 months |
| Field et al. 2007[64] | Unclear | Unclear | High | Low (groups received intervention or sham procedure) | Unclear | Low | Conflicting funding interest | Unclear |
| Freburger et al. 2010[56] | Low - varying permuted blocks stratified by class site | Low - concealed allocation | High | High (wait list control) | Low | Low | No | Unclear |
| Harvey et al. 2017[72] | Low - random numbers table | Low | Low | Low | Low | Low | Very small feasibility study | Unclear. Feasibility study |
| Jungquist et al. 2010[36] | Low | Unclear | High | Low (groups received intervention or sham procedure) | Low | Low | No | Low |
| Külcü et al. 2009[66] | Unclear | Unclear | High | High | Low | Low | No | High |
| Lami et al. 2018[48] | Unclear | Unclear | High | High (no intervention in control group) | High 28% lost to follow up | No | No | High |
| Lopez-Rodriguez et al. 2013[59] | Low - computerised number generator | Unclear | High but investigator blinded | Low (groups received intervention or sham procedure) | Unclear. ITT but 22.4% left during programme and some people attended <60% sessions | No | No | Unclear |
| Lu et al. 2017[60] | Low - computer-generated permutated block size 4 | Low - assignments by research staff not directly involved in trial | High | Low (groups received intervention or sham procedure) | Low 2/23 and 4/23 lost to follow up | Low | No | Low |
| Maddali Bongi et al. 2016[61] | Unclear | Unclear | High | Low (groups received intervention or sham procedure) | Low - 6 lost to follow up overall | Low | No | Unclear |
| Martinez et al. 2014[44] | Low - computerised number generator | Low risk - personnel blinded | High | Low (groups received intervention or sham procedure) | Low at post-treatment | Low risk | No | Low |
| McCrae et al. 2019[42] | Low - computer generated block size 6 | Low | High | High (wait list control) | Unclear 75% assessed at end intervention - 35% loss to follow-up at 6 months | Low | No | Unclear |
| Minetto et al. 2018[74] | High - pseudo-random procedure | High | High | High all patients received rehabilitation | Unclear | High | No | High |
| Miro et al. 2011[45] | Low - computer generated | Low | High | Low (groups received intervention or sham procedure) | Low | Low | No | Low |
| Murphy et al. 2019[69] | Low - statistical consultant generated randomisation schedule in blocks of 6 using SAS | Low | High - usual care group not blinded | Unclear (phone calls made to controls) | Low - ITT but higher drop out from intervention group than control | Unclear - Actiwatch results not reported because of difficulties obtaining data | Data reported in graphs - means (SDs) not reported | Unclear |
| Pearl et al. 1996[70] | Unclear | Unclear | Low | Low (groups received intervention or sham procedure) | High 26% did not complete study | Low | Unclear: Inclusion criteria age 21-65, but patients included age 19 | High |
| Pigeon et al. 2012[37] | Low - computer generated block randomisation in blocks of 2 | Unclear | High | High (wait list control) | Low. All randomised participants completed the study | Low | No | Unclear |
| Sanchez et al. 2012[46] | Low | Low | High | Low (groups received intervention or sham procedure) | Unclear but no loss to follow up reported | Low | No | Low |
| Smith et al. 2015[38] | Unclear | Unclear | Low | Low | Low | Low | No | Low |
| Smitherman et al. 2016[39] | Low - generated by statistician who had no contact with participants | Low - sequentially numbered, opaque sealed envelopes | High - therapists not blinded, participants not informed which treatment expected to be superior | Low - groups received intervention or sham procedure | Unclear - 22% lost to follow-up by 6 weeks after treatment. ITT analysis | Low | No | Low |
| Soares and Grossi 2002[53] | Unclear | Unclear | High - not blinded | High (wait list control) | Low for post-treatment | High - non-significant results not reported | No | Unclear |
| Tang et al. 2012[40] | Low - randomisation with pre-determined list | Unclear | High | High (wait list control) | Low - 0 at post-treatment 20% at longer follow up | Unclear - not registered so unclear | No | Unclear |
| Van Gordon et al. 2017[52] | Low - participant pseudonyms were placed into a bowl and selected one at a time | Low | Low - participants blinded to allocation | Low (both groups received either an intervention or a sham procedure) | High - 28% lost to post-treatment | Unclear as study retrospectively registered | No | High |
| Vitiello et al. 2013[49] | Low - cluster RCT. Block randomisationwith computer algorithm | Low (cluster RCT) | Low - participants blinded to which of 3 study arms contained active treatments | Low - assessors blinded to assignment | Low | Low -all reported outcomes included in protocol paper | No | Low |
| Vitiello et al. 2009[41] | Unclear | Unclear | High | Low (groups received intervention or sham procedure) | Unclear no losses reported at end of intervention | Unclear | No | Unclear |
| Vitorino et al. 2006[63] | Low - randomisation table | Low | Low - patients evaluated by trained physiotherapists blind to randomisation. Researcher involved in treatment blind to all data | Low (groups received intervention or sham procedure) | Low. 47/50 completed study | Unclear | No | Low |
| Wiklund et al. 2018[50] | Low – Excel block randomization in groups of 30 patients | Unclear | High | Low (groups received intervention or sham procedure) | Overall loss to follow up 31% post-treatment | High- registry record includes outcomes not reported in the manuscript | No | High |
| Yeh et al. 2016[68] | Low - computer-generated simple randomization | Unclear | Unclear - participants blinded regarding the group but therapist not blinded | Low - data collected by trained collector blinded to group assignment | High - 25% loss to follow-up at 1 month and uneven between groups | High - in a cited feasibility study more outcomes collected | No | High |
