# Supplementary material for: The effectiveness of non-pharmacological sleep interventions for people with chronic pain: a systematic review and meta-analysis
Source: BMC Musculoskelet Disord. 2022 May 11;23:440. doi: 10.1186/s12891-022-05318-5 (PMC9092772; doi:10.1186/s12891-022-05318-5)
Supplement: Supplementary file 5 — Additional file 5. [file 12891_2022_5318_MOESM5_ESM.docx]

**Supplement. Table 5: Effect estimates**

| **Comparison (Int 1 vs Int 2)** | **Effect measure** | **Number of studies (Participants Intervention; comparator)** | **Effect Estimate (95% CI), p-value** | **I²** |
| --- | --- | --- | --- | --- |
| **1a. CBT-I vs no treatment, wait list or attentional control** | | | | |
| ***Sleep (various overall measures)*** | | | | |
| Post-treatment | SMD | 9 (203:183) | -1.23 (-1.76, -0.70), p<0.00001 | 80% |
| 3 months | SMD | 2 (65:68) | -0.80 (-2.17, 0.57), p=0.25 | 93% |
| 6 months | SMD | 3 (64:70) | -0.51 (-1.02, -0.01), p=0.04 | 41% |
| ***Sleep PSQI Subscale - sleep quality*** | | | | |
| Post-treatment | MD | 1(33:33) | -0.60 [-1.25, 0.05] |  |
| ***Sleep PSQI Subscale - sleep latency*** | | | | |
| Post-treatment | MD | 1(33:33) | -0.64 [-1.00, -0.28] |  |
| ***Sleep PSQI Subscale - duration*** | | | | |
| Post-treatment | MD | 1(33:33) | -0.67 [-0.97, -0.37] |  |
| ***Sleep PSQI Subscale - efficiency*** | | | | |
| Post-treatment | MD | 1(33:33) | 0.42 [-0.32, 1.16] |  |
| ***Sleep PSQI Subscale - disturbances*** | | | | |
| Post-treatment | MD | 1(33:33) | -0.20 [-1.43, 1.03] |  |
| ***Sleep PSQI Subscale - dysfunction*** | | | | |
| Post-treatment | MD | 1(33:33) | -0.35 [-0.83, 0.13] |  |
| ***Sleep - nocturnal activity (actigraph)*** | | | | |
| Post-treatment | MD | 1(32:28) | -5.10 [-8.19, -2.01] |  |
| 3 months | MD | 1(32:28) | -3.60 [-7.33, 0.13] |  |
| ***Sleep - onset latency (diary)*** | | | | |
| Post-treatment | MD | 7(168:160) | -17.42 [-28.41, -6.43], p=0.002 | 73% |
| 3 months | MD | 2(64:70) | -14.58 [-21.67, -7.50], p<0.0001 | 0% |
| 6 months | MD | 3(62:68) | -6.48 [-14.36, 1.40], p=0.11 | 0% |
| ***Sleep - efficiency (diary)*** | | | | |
| Post-treatment | MD | 8(174:164) | -9.86 [-14.06, -5.66], p<0.00001 | 67% |
| 3 months | MD | 2(64:70) | -9.36 [-15.10, -3.61], p=0.001 | 53% |
| 6 months | MD | 3(62:68) | -6.16 [-10.31, -2.01], p=0.004 | 46% |
| ***Sleep - wake after sleep onset (diary)*** | | | | |
| Post-treatment | MD | 7(168:160) | -31.12 [-43.55, -18.69], p<0.00001 | 71% |
| 3 months | MD | 2(64:70) | -29.15 [-55.10, -3.19], p=0.03 | 67% |
| 6 months | MD | 4(85:96) | -18.58 [-30.57, -6.58],p=0.002 | 51% |
| ***Sleep - Awakenings (diary)*** | | | | |
| Post-treatment | MD | 2(51:37) | -0.23 [-0.88, 0.42], p=0.48 | 0% |
| 3 months | MD | 1(32:28) | -1.20 [-2.28, -0.12] |  |
| ***Sleep - Total sleep time (diary)*** | | | | |
| Post-treatment | MD | 8(174:164) | -6.70 [-31.98, 18.58], p=0.60 | 58% |
| 3 months | MD | 2(64:70) | -22.10 [-69.11, 24.91], p=0.36 | 73% |
| 6 months | MD | 4(85:96) | -2.44 [-22.15, 17.26], p=0.81 | 0% |
| ***Sleep - onset latency (actigraph)*** | | | | |
| Post-treatment | MD | 3(80:81) | -3.89 [-14.55, 6.77], p=0.47 | 63% |
| 3 months | MD | 1(33:39) | 7.58 [-12.80, 27.96] |  |
| 6 months | MD | 3(56:64) | -4.15 [-9.97, 1.68], p=0.16 | 0% |
| ***Sleep - efficiency (actigraph)*** | | | | |
| Post-treatment | MD | 4(96:96) | -1.58 [-5.59, 2.42], p=0.44 | 50% |
| 3 months | MD | 1(33:39) | -2.00 [-9.01, 5.01] |  |
| 6 months | MD | 3(55:65) | -2.17 [-5.42, 1.09], p=0.19 | 0% |
| ***Sleep - wake after sleep onset (actigraph)*** | | | | |
| Post-treatment | MD | 3(80:81) | -13.12 [-24.39, -1.85], p=0.02 | 0% |
| 3 months | MD | 1(33:39) | -17.03 [-44.62, 10.56] |  |
| 6 months | MD | 3(56:64) | -8.31 [-20.16, 3.53], p=0.17 | 0% |
| ***Sleep - Total sleep time (actigraph)*** | | | | |
| Post-treatment | MD | 4(96:96) | 13.66 [-18.58, 45.91], p=0.41 | 51% |
| 3 months | MD | 1(33:39) | 5.65 [-32.05, 43.35] |  |
| 6 months | MD | 3(56:64) | 17.50 [-9.93, 44.93], p=0.21 | 11% |
| ***Sleep - onset latency (polysomnograph)*** | | | | |
| Post-treatment | MD | 2(65:74) | 5.89 [-9.16, 20.93], p=0.44 | 0% |
| 3 months | MD | 1(30:38) | -8.75 [-18.58, 1.08] |  |
| 6 months | MD | 2(56:59) | -5.24 [-23.33, 12.84], p=0.57 | 0% |
| ***Sleep - efficiency (polysomnograph)*** | | | | |
| Post-treatment | MD | 2(65:74) | -5.06 [-9.55, -0.57], p=0.03 | 0% |
| 3 months | MD | 1(30:38) | -1.00 [-6.74, 4.74] |  |
| 6 months | MD | 2(56:59) | -4.39 [-9.11, 0.33], p=0.07 | 0% |
| ***Sleep - wake after sleep onset (polysomnograph)*** | | | | |
| Post-treatment | MD | 2(65:74) | -33.91 [-51.82, -16.00], p=0.0002 | 0% |
| 3 months | MD | 1(31:38) | 8.20 [-17.69, 34.09] |  |
| 6 months | MD | 2(56:59) | -22.45 [-41.93, -2.97], p=0.02 | 0% |
| ***Sleep - Total sleep time (polysomnograph)*** | | | | |
| Post-treatment | MD | 2(65:74) | 16.41 [-23.60, 56.42], p=0.42 | 29% |
| 3 months | MD | 1(31:38) | -0.67 [-35.14, 33.80] |  |
| 6 months | MD | 2(56:59) | 18.63 [-12.36, 49.62], p=0.24 | 0% |
| ***Sleep - Epworth Sleepiness Scale*** | | | | |
| Post-treatment | MD | 2(22:19) | -0.58 [-3.30, 2.14], p=0.68 | 0% |
| ***Pain (various instruments)*** | | | | |
| Post-treatment only | SMD | 9(192:178) | -0.24 [-0.45, -0.03], p=0.02 | 0% |
| 3 months | SMD | 3(83:83) | -0.31 [-0.69, 0.07], p=0.11 | 31% |
| 6 months | SMD | 3(66:70) | 0.07 [-0.27, 0.41], p=0.68 | 0% |
| ***General psychological health*** | | | | |
| Post-treatment (POMS) | MD | 1(15:9) | -15.50 [-19.99, -11.01] |  |
| 6 months (POMS) | MD | 1(6:7) | -20.30 [-29.06, -11.54] |  |
| ***Anxiety*** | | | | |
| Post-treatment | SMD | 3(53:53) | -0.54 [-1.01, -0.06], p=0.03 | 28% |
| 3 months (GAD-7) | MD | 1(16:15) | -0.60 [-4.02, 2.82] |  |
| 6 months (STAI) | MD | 1(24:23) | -5.80 [-13.64, 2.04] |  |
| ***Depression*** | | | | |
| Post-treatment | SMD | 6(123:113) | -0.57 [-1.05, -0.08], p=0.02 | 65% |
| 3 months | SMD | 2(48:43) | -0.37 [-0.79, 0.05], p=0.08 | 0% |
| 6 months (BDI) | MD | 1(24:23) | -6.79 [-13.54, -0.04] |  |
| **1b. Brief cognitive behavioural therapy and sleep hygiene versus wait list control** | | | | |
| ***Sleep PSQI Subscale - sleep quality*** | | | | |
| Post-treatment | MD | 1(44:41) | -0.10 [-0.42, 0.22] |  |
| ***Sleep - latency (diary)*** | | | | |
| Post-treatment | MD | 1(44:41) | -24.50 [-43.18, -5.82] |  |
| ***Sleep - Awakenings (diary)*** | | | | |
| Post-treatment | MD | 1(44:41) | -0.10 [-2.26, 2.06] |  |
| ***Sleep - Total sleep time (diary)*** | | | | |
| Post-treatment | MD | 1(44:41) | -18.00 [-52.82, 16.82] |  |
| **1c. CBT-I versus sleep hygiene** | | | | |
| ***Sleep (various overall measures)*** | | | | |
| Post-treatment | SMD | 3(65:64) | -0.25 [-0.82, 0.33], p=0.40 | 61% |
| 3 months (PSQI) | MD | 1(29:22) | -2.18 [-4.28, -0.08] |  |
| 6 months | SMD | 2(33:27) | -0.12 [-0.84, 0.59], p=0.73 | 34% |
| ***Sleep PSQI Subscale - sleep quality*** | | | | |
| Post-treatment | MD | 1(30:27) | -0.68 [-1.04, -0.32] |  |
| 3 months | MD | 1(29:22) | -0.51 [-0.92, -0.10] |  |
| 6 months | MD | 1(27:20) | -0.11 [-0.64, 0.42] |  |
| ***Sleep PSQI Subscale - sleep latency*** | | | | |
| Post-treatment | MD | 1(30:27) | 0.06 [-0.46, 0.58] |  |
| 3 months | MD | 1(29:22) | 0.11 [-0.44, 0.66] |  |
| 6 months | MD | 1(27:20) | 0.28 [-0.34, 0.90] |  |
| ***Sleep PSQI Subscale – duration*** | | | | |
| Post-treatment | MD | 1(30:27) | -0.26 [-0.73, 0.21] |  |
| 3 months | MD | 1(29:22) | -0.31 [-0.81, 0.19] |  |
| 6 months | MD | 1(27:20) | -0.12 [-0.68, 0.44] |  |
| ***Sleep PSQI Subscale – efficiency*** | | | | |
| Post-treatment | MD | 1(30:27) | -0.64 [-1.20, -0.08] |  |
| 3 months | MD | 1(29:22) | -0.37 [-0.96, 0.22] |  |
| 6 months | MD | 1(27:20) | -0.17 [-0.83, 0.49] |  |
| ***Sleep PSQI Subscale – disturbances*** | | | | |
| Post-treatment | MD | 1(30:27) | -0.47 [-0.81, -0.13] |  |
| 3 months | MD | 1(29:22) | -0.56 [-0.85, -0.27] |  |
| 6 months | MD | 1(27:20) | -0.26 [-0.58, 0.06] |  |
| ***Sleep - latency (diary)*** | | | | |
| Post-treatment | MD | 1(15:17) | 1.90 [-6.81, 10.61] |  |
| 6 months | MD | 1(6:7) | -0.90 [-10.36, 8.56] |  |
| ***Sleep - efficiency (diary)*** | | | | |
| Post-treatment | MD | 1(15:17) | -3.30 [-7.17, 0.57] |  |
| 6 months | MD | 1(6:7) | -3.70 [-8.00, 0.60] |  |
| ***Sleep - wake after sleep onset (diary)*** | | | | |
| Post-treatment | MD | 1(15:17) | -16.40 [-31.74, -1.06] |  |
| 6 months | MD | 1(6:7) | -16.50 [-33.60, 0.60] |  |
| ***Sleep - Total sleep time (diary)*** | | | | |
| Post-treatment | MD | 1(15:17) | -8.40 [-46.80, 30.00] |  |
| 6 months | MD | 1(6:7) | -15.10 [-52.88, 22.68] |  |
| ***Sleep - latency (actigraph)*** | | | | |
| Post-treatment | MD | 1(15:17) | -2.30 [-8.07, 3.47] |  |
| 6 months | MD | 1(6:7) | -4.40 [-13.08, 4.28] |  |
| ***Sleep - efficiency (actigraph)*** | | | | |
| Post-treatment | MD | 1(15:17) | -2.60 [-5.97, 0.77] |  |
| 6 months | MD | 1(6:7) | -2.20 [-6.23, 1.83] |  |
| ***Sleep - wake after sleep onset (actigraph)*** | | | | |
| Post-treatment | MD | 1(15:17) | -10.90 [-25.56, 3.76] |  |
| 6 months | MD | 1(6:7) | -6.60 [-23.53, 10.33] |  |
| ***Sleep - Total sleep time (actigraph)*** | | | | |
| Post-treatment | MD | 1(15:17) | -8.10 [-41.38, 25.18] |  |
| 6 months | MD | 1(6:7) | -6.70 [-35.57, 22.17] |  |
| ***Sleep - latency (polysomnograph)*** | | | | |
| Post-treatment | MD | 1(13:13) | 9.00 [-16.46, 34.46] |  |
| ***Sleep - efficiency (polysomnograph)*** | | | | |
| Post-treatment | MD | 1(13:13) | 1.81 [-3.71, 7.33] |  |
| ***Sleep - wake after sleep onset (polysomnograph)*** | | | | |
| Post-treatment | MD | 1(13:13) | 5.00 [-11.57, 21.57] |  |
| ***Sleep - Total sleep time (polysomnograph)*** | | | | |
| Post-treatment | MD | 1(13:13) | 5.00 [-76.26, 86.26] |  |
| ***Pain (various instruments)*** | | | | |
| Post-treatment only | SMD | 3(65:64) | -0.51 [-1.17, 0.15], p=0.13) | 70% |
| 3 months (McGill) | MD | 1(29:22) | -0.79 [-1.85, 0.27] |  |
| Longest follow up (3 months or longer) | SMD | 2(33:27) | 0.54 [-1.30, 2.38], p=0.57 | 85% |
| ***Health related quality of life*** |  |  |  |  |
| Post-treatment | SMD | 2(50:47) | -0.79 [-1.20, -0.37], p=0.0002 | 0% |
| 3 months (FIQ) | MD | 1(29:22) | -9.49 [-18.30, -0.68] |  |
| 6 months (FIQ) | MD | 1(27:20) | -9.78 [-19.42, -0.14] |  |
| ***General psychological health*** | | | | |
| Post-treatment (SF-36 MH) | MD | 1(15:17) | -0.90 [-4.94, 3.14] |  |
| 6 months (SF-36 MH) | MD | 1(6:7) | 0.30 [-6.58, 7.18] |  |
| ***Anxiety*** | | | | |
| Post-treatment | SMD | 2(50:47) | -0.32 [-0.72, 0.08], p=0.12 | 0% |
| 3 months (SCL-90-R) | MD | 1(29:22) | -0.15 [-0.66, 0.36] |  |
| 6 months (SCL-90-R) | MD | 1(27:20) | -0.05 [-0.54, 0.44] |  |
| ***Depression*** | | | | |
| Post-treatment | SMD | 2(50;47) | -0.61 [-1.05, -0.18], p=0.006 | 11% |
| 3 months (SCL-90-R) | MD | 1(29:22) | -0.64 [-1.10, -0.18] |  |
| 6 months (SCL-90-R) | MD | 1(27:20) | -0.33 [-0.84, 0.18] |  |
| **1d. CBT-IP versus no treatment, wait list or attentional control** | | | | |
| ***Sleep (various overall measures)*** | | | | |
| Post-treatment | SMD | 4(180:192) | -0.79 [-1.58, 0.00], p=0.05 | 88% |
| 3 months | SMD | 2(56:56) | -0.41 [-1.96, 1.15], p=0.61 | 94% |
| 6 months | SMD | 2(142:150) | -0.76 [-1.85, 0.33], p=0.17 | 92% |
| ***Sleep PSQI Subscale - sleep quality*** | | | | |
| Post-treatment | MD | 1(27:36) | 0.07 [-0.34, 0.48] |  |
| 3 months | MD | 1(22:26) | 0.30 [-0.12, 0.72] |  |
| ***Sleep PSQI Subscale - sleep latency*** | | | | |
| Post-treatment | MD | 1(27:36) | -0.26 [-0.77, 0.25] |  |
| 3 months | MD | 1(22:26) | 0.14 [-0.49, 0.77] |  |
| ***Sleep PSQI Subscale – disturbances*** | | | | |
| Post-treatment | MD | 1(27:36) | 0.13 [-0.25, 0.51] |  |
| 3 months | MD | 1(22:26) | 0.23 [-0.18, 0.64] |  |
| ***Sleep PSQI Subscale - efficiency*** | | | | |
| Post-treatment | MD | 1(27:36) | -0.11 [-0.70, 0.48] |  |
| 3 months | MD | 1(22:26) | 0.05 [-0.59, 0.69] |  |
| ***Sleep PSQI Subscale - duration*** | | | | |
| Post-treatment | MD | 1(27:36) | 0.07 [-0.40, 0.54] |  |
| 3 months | MD | 1(22:26) | 0.19 [-0.34, 0.72] |  |
| ***Sleep PSQI Subscale - dysfunction*** | | | | |
| Post-treatment | MD | 1(27:36) | 0.44 [-0.09, 0.97] |  |
| 3 months | MD | 1(22:26) | 0.70 [0.13, 1.27] |  |
| ***Sleep - efficiency (diary)*** | | | | |
| Post-treatment | MD | 1(6:4) | -7.20 [-15.96, 1.56] |  |
| ***Sleep - Total sleep time (diary)*** | | | | |
| Post-treatment | MD | 2(40:34) | -61.58 [-105.25, -17.91], p=0.006 | 0% |
| 3 months | MD | 1(34:30) | -84.00 [-126.40, -41.60] |  |
| 6 months | MD | 1(34:30) | -66.00 [-108.40, -23.60] |  |
| ***Sleep - efficiency (actigraph)*** |  |  |  |  |
| Post-treatment | MD | 1(96;115) | 2.64 [-1.39, 6.67] |  |
| 6 months | MD | 1(94:107) | 2.64 [-1.63, 6.91] |  |
| **Sleep - Epworth Sleepiness Scale** | | | | |
| Post-treatment | MD | 1(6:4) | -3.80 [-10.92, 3.32] |  |
| **Pain (various instruments)** | | | | |
| Post-treatment only | SMD | 4(180:182) | -0.13 (-0.36, 0.10), p=0.28 | 10% |
| 3 months | SMD | 2(56:56) | -0.48 [-0.86, -0.10], p=0.01 | 0% |
| 6 months | SMD | 3(161:395) | -0.25 [-0.62, 0.13], p=0.20 | 58% |
| ***Health related quality of life*** | | | | |
| Post-treatment | SMD | 2(58:65) | -1.77 [-5.33, 1.78], p=0.33 | 98% |
| 3 months | SMD | 2(54:49) | -1.84 [-5.90, 2.22], p=037 | 98% |
| 6 months (FIQ) | MD | 1(26:22) | -18.00 [-20.05, -15.95] |  |
| ***General psychological health*** | | | | |
| Post-treatment (HADS total) | MD | 1(31:29) | -7.00 [-7.74, -6.26] |  |
| 3 months (HADS total) | MD | 1(32:23) | -6.90 [-7.63, -6.17] |  |
| 6 months (HADS total) | MD | 1(26:22) | -8.00 [-8.77, -7.23] |  |
| ***Anxiety*** | | | | |
| Post-treatment (SCL-90-R) | MD | 1(27:36) | 0.31 [-0.19, 0.81] |  |
| 3 months (SCL-90-R) | MD | 1(22:26) | 0.44 [-0.05, 0.93] |  |
| ***Depression*** | | | | |
| Post-treatment | SMD | 2(33:42) | 0.14 [-0.54, 0.83], p=0.68 | 33% |
| 3 months (SCL-90-R) | MD | 1(22:26) | 0.55 [0.03, 1.07] |  |
| **1e. CBT-IP versus CBT-P control** | | | | |
| **Sleep (various overall measures)** | | | | |
| Post-treatment | SMD | 3(146:150) | -0.47 [-1.19, 0.25], p=0.20 | 75% |
| 3 months (PSQI) | MD | 1(22:24) | -0.22 [-2.49, 2.05] |  |
| ***Sleep PSQI Subscale - sleep quality*** | | | | |
| Post-treatment | MD | 1(27:28) | 0.07 [-0.38, 0.52] |  |
| 3 months | MD | 1(22:24) | 0.16 [-0.29, 0.61] |  |
| ***Sleep PSQI Subscale - sleep latency*** | | | | |
| Post-treatment | MD | 1(27:28) | 0.03 [-0.54, 0.60] |  |
| 3 months | MD | 1(22:24) | 0.08 [-0.48, 0.64] |  |
| ***Sleep PSQI Subscale - duration*** | | | | |
| Post-treatment | MD | 1(27:28) | -0.25 [-0.71, 0.21] |  |
| 3 months | MD | 1(22:24) | 0.13 [-0.39, 0.65] |  |
| ***Sleep PSQI Subscale - efficiency*** | | | | |
| Post-treatment | MD | 1(27:28) | 0.03 [-0.61, 0.67] |  |
| 3 months | MD | 1(22:24) | 0.19 [-0.47, 0.85] |  |
| ***Sleep PSQI Subscale - disturbances*** | | | | |
| Post-treatment | MD | 1(27:28) | -0.11 [-0.47, 0.25] |  |
| 3 months | MD | 1(22:24) | -0.33 [-0.72, 0.06] |  |
| ***Sleep PSQI Subscale - dysfunction*** | | | | |
| Post-treatment | MD | 1(27:28) | -0.02 [-0.53, 0.49] |  |
| 3 months | MD | 1(22:24) | -0.14 [-0.63, 0.35] |  |
| ***Sleep - efficiency (diary)*** | | | | |
| Post-treatment | MD | 1(6:5) | -7.20 [-16.15, 1.75] |  |
| ***Sleep - efficiency (actigraph)*** | | | | |
| Post-treatment | MD | 1(96:115) | -2.64 [-6.68, 1.40] |  |
| 6 months | MD | 1(94:107) | -2.64 [-6.92, 1.64] |  |
| ***Sleep - Total sleep time (diary)*** | | | | |
| Post-treatment | MD | 1(6:5) | -47.70 [-127.90, 32.50] |  |
| ***Sleep - Epworth Sleepiness Scale*** | | | | |
| Post-treatment | MD | 1(6:5) | -0.20 [-3.49, 3.09] |  |
| ***Pain (various instruments)*** | | | | |
| Post-treatment | SMD | 3(146:150) | -0.03 [-0.26, 0.20], p=0.78 | 0% |
| 3 months (McGill) | MD | 1(22:26) | -0.4(-0.9-0.58 [-1.44, 0.28],0.2) |  |
| 6 months (Graded Chronic Pain Scale) | MD | 1(108:111) | -0.17 [-1.02, 0.68] |  |
| ***Heath related quality of life*** | | | | |
| Post-treatment | SMD | 2(139;145) | 0.03 [-0.20, 0.26], p=0.81 | 0% |
| 3 months (SCL-90-R) | MD | 1(22:24) | 3.20 [-5.13, 11.53] |  |
| 6 months (AIMS-2) | MD | 1(107:111) | 0.26 [-0.73, 1.25] |  |
| ***Anxiety*** | | | | |
| Post-treatment | MD | 1(27:28) | -0.03 [-0.56, 0.50] |  |
| 3 months | MD | 1(22:24) | 0.02 [-0.56, 0.60] |  |
| ***Depression*** | | | | |
| Post-treatment | SMD | 2(33:33) | -0.34 [-1.05, 0.37], p=0.35 | 28% |
| 3 months | MD | 1(22:24) | -0.09 [-0.64, 0.46] |  |
| **1f. CBT-IP plus hypnosis versus CBT-IP alone** | | | | |
| ***Sleep (MOS Sleep problems index)*** | | | | |
| Post-treatment | MD | 1(29:34) | -0.90 [-4.64, 2.84] |  |
| 3 months | MD | 1(29:34) | -0.60 [-5.32, 4.12] |  |
| 6 months | MD | 1(29:34) | 0.30 [-4.00, 4.60] |  |
| ***Sleep - Total sleep time (diary)*** | | | | |
| Post-treatment | MD | 1(29:34) | -6.00 [-55.89, 43.89] |  |
| 3 months | MD | 1(29:34) | 0.00 [-42.40, 42.40] |  |
| 6 months | MD | 1(29:34) | -12.00 [-54.40, 30.40] |  |
| ***Pain*** | | | | |
| Post-treatment only (NRS pain) | MD | 1(29:34) | -0.30 [-1.13, 0.53] |  |
| 3 months (NRS pain) | MD | 1(29:34) | -0.20 [-1.14, 0.74] |  |
| 6 months (NRS pain) | MD | 1(29:34) | -0.10 [-1.08, 0.88] |  |
| ***Health related quality of life*** | | | | |
| Post-treatment only (FIQ) | MD | 1(29:34) | -3.20 [-4.89, -1.51] |  |
| 3 months (FIQ) | MD | 1(26:32) | -1.70 [-3.50, 0.10] |  |
| 6 months (FIQ) | MD | 1(23:26) | 4.50 [2.45, 6.55] |  |
| ***General psychological health*** | | | | |
| Post-treatment only (HADS total) | MD | 1(27:31) | -4.40 [-5.15, -3.65] |  |
| 3 months (HADS total) | MD | 1(26:32) | -2.20 [-2.90, -1.50] |  |
| 6 months (HADS total) | MD | 1(23:26) | -1.50 [-2.26, -0.74] |  |
| **1g. Acceptance and Commitment Therapy based stress management versus control** | | | | |
| ***Sleep*** | | | | |
| Post-treatment (Insomnia Severity Index) | MD | 1(64:61) | 0.00 [-2.45, 2.45] |  |
| 6 months (Insomnia Severity Index) | MD | 1(54:55) | 1.25 [-1.28, 3.78] |  |
| ***Pain*** | | | | |
| Post-treatment (NRS pain) | MD | 1(64:61) | 0.07 [-0.65, 0.79] |  |
| 6 months (NRS pain) | MD | 1(55:54) | 0.65 [-0.17, 1.47] |  |
| ***Anxiety*** | | | | |
| Post-treatment (HADS anxiety) | MD | 1(64:61) | -0.83 [-2.37, 0.71] |  |
| 6 months (HADS anxiety) | MD | 1(55:54) | 0.49 [-0.93, 1.91] |  |
| ***Depression*** | | | | |
| Post-treatment (HADS depression) | MD | 1(64:61) | 0.37 [-0.93, 1.67] |  |
| 6 months (HADS depression) | MD | 1(55:54) | 0.73 [-0.65, 2.11] |  |
| **1h. Acceptance and Commitment Therapy based stress management versus exercise** | | | | |
| ***Sleep*** | | | | |
| Post-treatment (Insomnia Severity Index) | MD | 1(64:75) | 1.76 [-0.40, 3.92] |  |
| 6 months (Insomnia Severity Index) | MD | 1(54:66) | 1.15 [-1.26, 3.56] |  |
| ***Pain*** | | | | |
| Post-treatment (NRS pain) | MD | 1(64:75) | 0.95 [0.24, 1.66] |  |
| 6 months (NRS pain) | MD | 1(54:66) | 0.43 [-0.40, 1.26] |  |
| ***Anxiety*** | | | | |
| Post-treatment (HADS anxiety) | MD | 1(64:75) | 0.18 [-1.27, 1.63] |  |
| 6 months (HADS anxiety) | MD | 1(54:66) | -0.45 [-1.90, 1.00] |  |
| ***Depression*** | | | | |
| Post-treatment (HADS depression) | MD | 1(64:75) | 0.57 [-0.70, 1.84] |  |
| 6 months (HADS depression) | MD | 1(54:66) | 0.43 [-0.84, 1.70] |  |
| **1i. Mindfulness versus control** | | | | |
| ***Sleep quality overall*** | | | | |
| Post-treatment | SMD | 2(105:92) | -0.41 [-0.72, -0.11], p=0.008 | 14% |
| 3 months (SSQ) | MD | 1(51:40) | -1.10 [-2.48, 0.28] |  |
| 6 months (PSQI) | MD | 1(45:40) | -2.28 [-3.48, -1.08] |  |
| ***Pain*** | | | | |
| Post-treatment | SMD | 2(105:92) | -0.35 [-0.63, -0.06], p=0.02 | 0% |
| 3 months (VAS pain) | MD | 1(51:40) | 0.10 [-9.59, 9.79] |  |
| 6 months (McGill) | MD | 1(45:40) | -2.55 [-4.54, -0.56] |  |
| ***Health related quality of life*** | | | | |
| Post-treatment | SMD | 2(105:92) | -0.28 [-0.81, 0.26], p=0.31 | 72% |
| 3 months (FIQ) | MD | 1(51:40) | 0.00 [-0.31, 0.31] |  |
| 6 months (FIQ) | MD | 1(45:40) | -6.71 [-11.01, -2.41] |  |
| ***General psychological health*** | | | | |
| Post-treatment (DASS) | MD | 1(54:52) | -3.34 [-5.08, -1.60] |  |
| 6 months (DASS) | MD | 1(45:40) | -4.50 [-6.75, -2.25] |  |
| **1j. Relaxation versus waitlist control** | | | | |
| ***Sleep quality overall*** | | | | |
| Post-treatment (Karolinska Sleep Quality) | MD | 1(18:17) | -0.10 [-0.67, 0.47] |  |
| ***Pain*** | | | | |
| Post-treatment (McGill) | MD | 1(18:17) | -1.60 [-23.85, 20.65] |  |
| ***Health related quality of life*** |  |  |  |  |
| Post-treatment (FIQ) | MD | 1(18:17 | -0.11 [-0.48, 0.26] |  |
| **Relaxation versus education** |  |  |  |  |
| Post-treatment (Karolinska Sleep Quality) | MD | 1(18:18) | -0.23 [-0.80, 0.34] |  |
| 6 months (Karolinska Sleep Quality) | MD | 1(14:15) | -0.87 [-1.69, -0.05] |  |
| ***Pain*** |  |  |  |  |
| Post-treatment (McGill) | MD | 1(18:18) | -5.50 [-30.73, 19.73] |  |
| 6 months (McGill) | MD | 1(14:15) | -3.08 [-26.81, 20.65] |  |
| ***Health related quality of life*** |  |  |  |  |
| Post-treatment (FIQ) | MD | 1(18:18) | -0.41 [-0.89, 0.07] |  |
| 6 months (FIQ) | MD | 1(14:15) | -0.03 [-0.58, 0.52] |  |
| **2. Sleep hygiene versus standard care/waitlist control** | | | | |
| ***Sleep quality overall (various measures)*** | | | | |
| Post-treatment | SMD | 2(35:26) | -0.68 [-2.37, 1.02], p=0.43 | 89% |
| 6 months (ISQ) | MD | 1(7:7) | -21.60 [-33.81, -9.39] |  |
| ***Sleep - latency (diary)*** | | | | |
| Post-treatment | MD | 1(17:9) | -0.80 [-12.98, 11.38] |  |
| 6 months | MD | 1(7:7) | -13.10 [-40.88, 14.68] |  |
| ***Sleep - efficiency (diary)*** | | | | |
| Post-treatment | MD | 1(17:9) | -1.40 [-7.16, 4.36] |  |
| 6 months | MD | 1(7:7) | -4.00 [-8.46, 0.46] |  |
| ***Sleep – waking after sleep onset (diary)*** | | | | |
| Post-treatment | MD | 1(17:9) | -15.20 [-39.66, 9.26] |  |
| 6 months | MD | 1(7:7) | -11.00 [-33.18, 11.18] |  |
| ***Sleep - Total sleep time (diary)*** | | | | |
| Post-treatment | MD | 1(17:9) | 7.70 [-38.38, 53.78] |  |
| 6 months | MD | 1(7:7) | -8.30 [-67.16, 50.56] |  |
| ***Sleep - latency (actigraph)*** | | | | |
| Post-treatment | MD | 1(17:9) | -5.80 [-15.45, 3.85] |  |
| 6 months | MD | 1(7:7) | -1.10 [-11.52, 9.32] |  |
| ***Sleep - efficiency (actigraph)*** | | | | |
| Post-treatment | MD | 1(17:9) | -2.80 [-9.47, 3.87] |  |
| 6 months | MD | 1(7:7) | -1.00 [-6.42, 4.42] |  |
| ***Sleep – waking after sleep onset (actigraph)*** | | | | |
| Post-treatment | MD | 1(17:9) | -12.60 [-40.94, 15.74] |  |
| 6 months | MD | 1(7:7) | -9.50 [-31.58, 12.58] |  |
| ***Sleep - Total sleep time (actigraph)*** | | | | |
| Post-treatment | MD | 1(17:9) | 7.10 [-49.54, 63.74] |  |
| 6 months | MD | 1(7:7) | 24.30 [-22.34, 70.94] |  |
| ***Pain (various instruments)*** | | | | |
| Post-treatment | SMD | 2(35:26) | -0.22 [-0.93, 0.49], p=0.54 | 45% |
| 6 months (McGill) | MD | 1(7:7) | -11.70 [-23.97, 0.57] |  |
| ***Health related quality of life*** |  |  |  |  |
| Post-treatment (FIQ) | MD | 1(18:17) | 0.01 [-0.42, 0.44] |  |
| ***General psychological health*** |  |  |  |  |
| Post-treatment (POMS) | MD | 1(17:9) | -14.60 [-19.88, -9.32] |  |
| 6 months (POMS) | MD | 1(7:7) | -20.60 [-30.06, -11.14] |  |
| **3a. Group-based exercise versus control** | | | | |
| ***Sleep (various overall measures)*** | | | | |
| Post-treatment | SMD | 3(266:240) | -0.10 [-0.31, 0.12] p=0.39 | 27% |
| 6 months (ISI) | MD | 1(66:55) | 0.10 [-2.61, 2.81] |  |
| ***Pain (various overall measures)*** | | | | |
| Post-treatment | SMD | 3(142:125) | -0.52 [-0.76, -0.27], p<0.00001 | 0% |
| 6 months (NRS pain) | MD | 1(59:54) | 0.02 [-0.35, 0.39] |  |
| ***Health related quality of life*** |  |  |  |  |
| Post-treatment (HAQ) | MD | 1(40:38) | -0.30 [-0.57, -0.03] |  |
| ***General psychological health*** | | | | |
| Post-treatment (SF-36 MH) | MD | 1(27:26) | -1.57 [-6.43, 3.29] |  |
| ***Anxiety*** | | | | |
| Post-treatment | SMD | 2(91:81) | -0.51 [-1.43, 0.40], p=0.27 | 86% |
| 6 months (HADS) | MD | 1(58:47) | 1.32 [-0.36, 3.00] |  |
| ***Depression*** | | | | |
| Post-treatment | SMD | 2(91:81) | -0.41 [-1.26, 0.44], p=0.35 | 85% |
| 6 months (HADS) | MD | 1(58:47) | 0.65 [-0.71, 2.01] |  |
| **3b. Home based walking programme versus supervised exercise class** | | | | |
| ***Sleep quality*** | | | | |
| Post-treatment (PSQI - change score) | MD | 1(17:13) | 0.48 [-2.15, 3.11] |  |
| 3 months (PSQI - change score) | MD | 1(15:13) | -1.09 [-3.98, 1.80] |  |
| ***Pain*** | | | | |
| Post-treatment (NRS average back pain – change score) | MD | 1(17:13) | 1.28 [-0.65, 3.21] |  |
| 3 months (NRS average back pain – change score) | MD | 1(16:13) | 2.18 [0.07, 4.29] |  |
| ***General psychological health*** | | | | |
| Post-treatment (SF-36 MH) | MD | 1(17:14) | -5.69 [-12.38, 1.00] |  |
| 3 months (SF-36 MH) | MD | 1(16:13) | -3.63 [-11.16, 3.90] |  |
| ***Anxiety*** | | | | |
| Post-treatment (HADS) | MD | 1(17:14) | -0.16 [-1.98, 1.66] |  |
| 3 months (HADS) | MD | 1(16:13) | 1.16 [-1.32, 3.64] |  |
| ***Depression*** |  |  |  |  |
| Post-treatment (HADS) | MD | 1(17:14) | -0.26 [-2.12, 1.60] |  |
| 3 months (HADS) | MD | 1(16:13) | 0.32 [-2.64, 3.28] |  |
| **3c. Moderate aerobic exercise versus low intensity home-based exercise** | | | | |
| ***Sleep*** | | | | |
| Post-treatment (PSQI) | MD | 1(17:13) | -2.50 [-4.55, -0.45] |  |
| ***Sleep efficiency (actigraph)*** | | | | |
| Post-treatment | MD | 1(11:10) | -9.52 [-18.10, -0.94] |  |
| ***Waking after sleep onset (actigraph)*** | | | | |
| Post-treatment | MD | 1(11:10) | -54.00 [-85.26, -22.74] |  |
| ***Total sleep time (actigraph)*** | | | | |
| Post-treatment | MD | 1(11:10) | --52.40 [-100.90, -3.90] |  |
| **3d. Aquatic biodance versus stretching exercises** | | | | |
| ***Sleep quality*** | | | | |
| Post-treatment (PSQI) | MD | 1(38:38) | -6.38 [-7.44, -5.32] |  |
| ***Pain*** | | | | |
| Post-treatment | MD | 1(38:38) | -7.04 [-10.05, -4.03] |  |
| ***Health related quality of life*** | | | | |
| Post-treatment | MD | 1(38:38) | -13.81 [-27.30, -0.32] |  |
| ***Anxiety*** | | | | |
| Post-treatment | MD | 1(38:38) | -5.24 [-7.80, -2.68] |  |
| ***Depression*** | | | | |
| Post-treatment | MD | 1(38:38) | -6.21 [-10.61, -1.81] |  |
| **3e. Tai Ji Quan vs education** | | | | |
| ***Sleep quality*** | | | | |
| Post-treatment (PSQI change and endpoint) | MD | 2(45:45) | -0.78 [-2.31, 0.76], p=0.32 | 0% |
| ***Sleep (PSQI subscale - sleep quality)*** | | | | |
| Post-treatment | MD | 1(23:23) | 0.31 [-0.20, 0.82] |  |
| ***Sleep (PSQI subscale - sleep latency)*** | | | | |
| Post-treatment | MD | 1(23:23) | -0.17 [-0.83, 0.49] |  |
| ***Sleep (PSQI subscale - sleep duration)*** | | | | |
| Post-treatment | MD | 1(23:23) | -0.25 [-0.77, 0.27] |  |
| ***Sleep (PSQI subscale - sleep efficiency)*** | | | | |
| Post-treatment | MD | 1(23:23) | -0.75 [-1.43, -0.07] |  |
| ***Sleep (PSQI subscale - sleep disturbance)*** | | | | |
| Post-treatment | MD | 1(23:23) | -0.12 [-0.61, 0.37] |  |
| ***Sleep (PSQI subscale - sleep dysfunction)*** | | | | |
| Post-treatment | MD | 1(23:23) | 0.04 [-0.66, 0.74] |  |
| ***Sleep onset latency (diary)*** | | | | |
| Post-treatment | MD | 1(23:23) | -1.46 [-18.47, 15.55] |  |
| ***Total sleep time (diary)*** | | | | |
| Post-treatment | MD | 1(23:23) | -9.60 [-42.10, 22.90] |  |
| ***Sleep efficiency (diary)*** | | | | |
| Post-treatment | MD | 1(23:23) | -8.68 [-16.29, -1.07] |  |
| ***Pain*** | | | | |
| Post-treatment | MD | 1(22:22) | -2.09 [-4.59, 0.41] |  |
| ***General psychological health*** | | | | |
| Post-treatment (SF-36 MH) | MD | 1(23:23) | -4.92 [-11.29, 1.45] |  |
| ***Anxiety*** | | | | |
| Post-treatment (HADS | MD | 1(22:22) | 0.73 [-1.91, 3.37] |  |
| ***Depression*** | | | | |
| Post-treatment (HADS) | MD | 1(22:22) | 0.91 [-0.61, 2.43] |  |
| **4.1. Hydrotherapy versus Physiotherapy** | | | | |
| ***Total sleep time*** | | | | |
| Post-treatment | MD | 1(25:25) | 0.62 [1.27, -0.03] |  |
| ***Total nap time*** | | | | |
| Post-treatment | MD | 1(25:25) | -2.97 [-3.00, -2.94] |  |
| ***Health related quality of life*** | | | | |
| Post-treatment (SF-36 BP) | MD | 1(25:25) | 5.80 [-5.82, 17.42] |  |
| ***General psychological health*** | | | | |
| Post-treatment (SF-36 MH) | MD | 1(25:25) | 2.50 [-9.95, 14.95] |  |
| **4.2. Massage versus Relaxation** | | | | |
| ***Sleep disturbance*** | | | | |
| Post-treatment | MD | 1(15:15) | -3.50 [-17.75, 10.75] |  |
| ***Pain*** | | | | |
| Post-treatment (VAS) | MD | 1(15:15) | -1.30 [-2.76, 0.16] |  |
| ***General psychological health*** | | | | |
| Post-treatment (POMS) | MD | 1(15:15) | -1.40 [-6.22, 3.42] |  |
| ***Anxiety*** | | | | |
| Post-treatment (STAI) | MD | 1(15:15) | 1.10 [-4.28, 6.48] |  |
| **4.3. Physical therapy programme versus control** | | | | |
| ***Sleep*** | | | | |
| Post-treatment (ISI) | MD | 1(40:20) | -2.40 [-6.09, 1.29] |  |
| ***Pain*** | | | | |
| Post-treatment (VAS) | MD | 1(40:20) | -1.90 [-3.26, -0.54] |  |
| ***Health related quality of life*** | | | | |
| Post-treatment (FIQ) | MD | 1(40:20) | -16.70 [-24.57, -8.83] |  |
| **4.4. Pompage versus Control** | | | | |
| ***Sleep quality*** | | | | |
| Post-treatment (Sleep inventory) | MD | 1(7:8) | 0.74 [-0.48, 1.96] |  |
| ***Pain*** | | | | |
| Post-treatment (McGill) | MD | 1(7:8) | -7.80 [-20.18, 4.58] |  |
| **5.1. Auricular point acupressure versus sham** | | | | |
| **Sleep quality** | | | | |
| Post-treatment (change in global PSQI) | MD | 1(25:21) | -1.05 [-2.05, -0.05] |  |
| ***Perceived sleep quality*** | | | | |
| Post-treatment (change) | MD | 1(25:21) | -0.16 [-0.36, 0.04] |  |
| ***Sleep efficiency*** | | | | |
| Post-treatment (change) | MD | 1(25:21) | -0.10 [-0.51, 0.31] |  |
| ***Daytime disturbance*** | | | | |
| Post-treatment (change) | MD | 1(25:21) | -0.18 [-0.39, 0.03] |  |
| ***Pain*** | | | | |
| Post-treatment (change BPI worst pain) | MD | 1(25:21) | -2.86 [-4.31, -1.41] |  |
| **5.2. Bright light treatment versus sham** | | | | |
| ***Sleep quality*** | | | | |
| Post-treatment (Post Sleep Questionnaire) | MD | 1(14:14) | -0.50 [-2.02, 1.02] |  |
| ***Hours sleep per night*** | | | | |
| Post-treatment | MD | 1(14:14) | -0.20 [-1.31, 0.91] |  |
| ***Number awakenings per night*** | | | | |
| Post-treatment | MD | 1(14:14) | -0.20 [-1.39, 0.99] |  |
| ***Pain*** | | | | |
| Post-treatment (Post Sleep Questionnaire) | MD | 1(14:14) | -1.50 [-8.93, 5.93] |  |
| ***Anxiety*** | | | | |
| Post-treatment (FIQ anxiety) | MD | 1(14:14) | -1.50 [-15.97, 12.97] |  |
| ***Depression*** | | | | |
| Post-treatment (FIQ depression) | MD | 1(14:14) | 2.30 [-11.53, 16.13] |  |
| ***5.3. Foot reflexology versus control*** | | | | |
| ***Sleep quality*** | | | | |
| Post-treatment (PSQI) | MD | 1(30:30) | -5.87 [-7.55, -4.19] |  |
| ***Pain*** | | | | |
| Post-treatment (VAS pain) | MD | 1(30:30) | -2.70 [-3.18, -2.22] |  |
| **5.5. Magnetic mattress pad versus sham** | | | | |
| ***Sleep quality*** | | | | |
| Post-treatment (VAS sleep) | MD | 1(13:12) | -1.00 [-1.53, -0.47] |  |
| ***Pain*** | | | | |
| Post-treatment (VAS pain) | MD | 1(13:12) | -1.90 [-2.51, -1.29] |  |
| ***Health related quality of life*** | | | | |
| Post-treatment (FIQ-ADL) | MD | 1(13:12) | -6.30 [-7.72, -4.88] |  |

ADL Activities of daily living, AIMS2 Arthritis Impact Measurement Scale 2, BDI Beck Depression Inventory, BPI Brief Pain Inventory, CBT-I cognitive behavioural therapy for insomnia, CBT-IP cognitive behavioural therapy for insomnia and pain, CBT-P cognitive behavioural therapy for pain, CI confidence interval, DASS Depression Anxiety Stress Scales, FIQ Fibromyalgia Impact Questionnaire, GAD General Anxiety Disorder-7, HADS Hospital Anxiety and Depression Scale, ISI Insomnia Severity Index, MD mean difference, MOS Medical Outcomes Study, NRS Numeric Rating Scale, POMS Profile of Mood Sates, PSQI Pittsburgh Sleep Quality Index, SCL-90-R Symptom Checklist-90-Revised, SF-36 BP 36-Item Short Form Survey (SF-36) Bodily Pain, SF-36-MH 36-Item Short Form Survey (SF-36) Mental Health, SMD standardised mean difference, SSQ Stanford Sleep Questionnaire, STAI State-Trait Anxiety Inventory, VAS Visual Analogue Scale
